# Bacterial community analysis of treponeme-associated hoof disease in free-ranging elk (*Cervus canadensis*): evidence for a polybacterial etiology with geographic consistency

**DOI:** 10.1128/aem.00888-25

**Published:** 2025-10-03

**Authors:** Elizabeth W. Goldsmith, Kyle R. Taylor, Margaret A. Wild, Sushanta Deb, Tarah Sullivan, Eric Lofgren, Kyle R. Garrison, Gregory M. Schroeder, Carrington Hilson, Nicole L. Walrath, Julia D. Burco, Emma Lantz, Steven N. Winter, Devendra H. Shah

**Affiliations:** 1 Department of Veterinary Microbiology and Pathology, College of Veterinary Medicine, Washington State University 6760, Pullman, Washington, USA; 2 Washington Animal Disease Diagnostic Laboratory, College of Veterinary Medicine, Washington State University 6760, Pullman, Washington, USA; 3 Department of Crop and Soil Sciences, College of Agricultural, Human, and Natural Resource Sciences, Washington State University 6760, Pullman, Washington, USA; 4 Paul G. Allen School for Global Health, College of Veterinary Medicine, Washington State University 6760, Pullman, Washington, USA; 5 Washington Department of Fish and Wildlife 266120, Olympia, Washington, USA; 6 National Park Service, Wind Cave National Park 7133, Hot Springs, South Dakota, USA; 7 California Department of Fish and Wildlife, Northern Region 66621, Eureka, California, USA; 8 Wildlife Health Laboratory, Idaho Department of Fish and Game 115870, Eagle, Idaho, USA; 9 Wildlife Health and Population Laboratory, Oregon Department of Fish and Wildlife 190732, Corvallis, Oregon, USA; 10 Wildlife Health Laboratory, California Department of Fish and Wildlife 66621, Rancho Cordova, California, USA; 11 School of Veterinary Medicine, Texas Tech University 6177, Amarillo, Texas, USA; Universidad de los Andes, Bogotá, Colombia

**Keywords:** 16S rRNA gene amplicon sequencing, TAHD lesion categorization, *Mycoplasma*, pododermatitis, polybacterial etiology, spirochete, *Treponema*

## Abstract

Treponeme-associated hoof disease (TAHD) is an emerging infectious disease in free-ranging elk (*Cervus canadensis*) characterized by ulcerative and necrosuppurative pododermatitis with spirochete bacteria that leads to lameness and apparent increased mortality. While TAHD is hypothesized to have a polybacterial etiology, the causative agents remain poorly characterized, particularly across its geographic range. In this study, we developed a histologic categorization system for hoof lesions and employed 16S rRNA gene amplicon sequencing to characterize bacterial communities in samples from 129 free-ranging elk across regions with endemic or sporadic TAHD and where TAHD remains undetected. Differential abundance analysis revealed strong associations between TAHD-positive lesions and the bacterial genera *Treponema*, unidentified *Spirochaetaceae*, *Mycoplasma*, and *Fusobacterium*, along with their respective families and amplicon sequence variants. Many of these TAHD-associated operational taxonomic units (OTUs) were also more frequently detected at increased abundance in more severe, histologic lesions of pododermatitis. Correlation analysis demonstrated a strong positive association between *Treponema* and *Mycoplasma* in TAHD lesions, suggesting a more significant role of *Mycoplasma* in TAHD’s etiology than previously recognized. Additionally, we identified novel TAHD-associated OTUs, including *Corynebacterium freneyi-xerosis*, that warrant further investigation. Comparative analysis of TAHD-positive lesions from endemic and sporadic areas revealed minimal differences in the microbial community. These findings advance our understanding of the bacterial contributors to TAHD, highlighting putative pathogens for further investigation and as potential targets for diagnostic development.

**IMPORTANCE:**

While detection of *Treponema* is a hallmark of treponeme-associated hoof disease (TAHD), a comprehensive understanding of other bacterial contributors is necessary to improve diagnostic testing and inform control measures. Our study confirmed strong associations between *Spirochaetaceae* and TAHD lesions and revealed a previously underappreciated role of *Mycoplasma* in TAHD’s etiology. *Treponema* and *Mycoplasma* were significantly enriched in TAHD-positive lesions, absent from TAHD-negative tissues, and strongly and positively correlated with each other, suggesting a potential synergistic relationship. By developing and applying a histologic categorization system, we characterized shifts in bacterial communities as lesion severity progressed. Comparisons of TAHD-positive lesions from endemic and sporadic regions revealed minimal differences in the microbial composition, indicating strong geographic consistency. These findings enhance our understanding of TAHD’s etiology and provide a foundation for future research, including the development of improved diagnostic tests and targeted disease management strategies.

## INTRODUCTION

Treponeme-associated hoof disease (TAHD) is an emerging transmissible disease that causes lameness, debilitation, and apparent increased mortality in free-ranging elk (*Cervus canadensis*) (1–3). Gross lesions associated with TAHD typically include ulceration, overgrown hooves, undermining of the heel bulb, and sloughing of the hoof capsule (2). However, these lesions are not pathognomonic, as other hoof pathologies can present with similar gross lesions. Consequently, the current diagnosis of TAHD relies on histologic examination, where TAHD-positive lesions are characterized by ulcerative and necrosuppurative pododermatitis with argyrophilic (silver-stain positive) spirochete bacteria (2, 4).

Recent investigations utilizing 16S rRNA gene amplicon sequencing, selective bacterial culture, and amplicon PCR have revealed that *Treponema* spp. are associated with TAHD lesions (2–6). The role of *Treponema* spp. in polybacterial periodontal disease in humans has been well established (7–9). Similarly, *Treponema* spp. have been implicated as etiologic agents of bovine digital dermatitis (BDD), a global hoof disease of cattle (10, 11), as well as contagious ovine digital dermatitis (CODD) in sheep and digital dermatitis in goats, neither of which is known to overlap geographically with TAHD (12–16). *Treponema* spp. that are routinely associated with BDD and CODD lesions (11, 12, 17, 18) have been broadly classified into three phylogroups (19–21): *Treponema phagedenis*, *T. medium* (comprising *T. medium* and *T. vincentii*), and *T. pedis* (comprising *T. pedis*, *T. denticola*, and *T. putidum*). These *Treponema* phylogroups, along with novel genomospecies from *Spirochaetaceae* and *Treponemataceae* families, have also been routinely detected in the majority of TAHD lesions (4–6, 22), suggesting a potential role for both known and newly discovered spirochetes in the etiology of TAHD. Additionally, operational taxonomic units (OTUs) representing bacterial families such as *Mycoplasmataceae*, *Fusobacteriaceae*, and *Porphyromonadaceae* have been consistently detected in both BDD and TAHD lesions (4, 6, 23), supporting the hypothesis that, similar to BDD, TAHD most likely has a polybacterial etiology.

Previous investigations into bacterial communities associated with TAHD lesions have primarily focused on comparing elk hooves based on gross lesions with some cases confirmed through broad histologic lesion descriptions and detection of argyrophilic spirochetes (2, 4, 6). Studies of BDD lesions in cattle have shown that interpretations of grossly detected hoof abnormalities are not always validated by subsequent histologic assessment (10, 24). These findings underscore the need for a systematic approach to histologic assessment of TAHD lesions and characterization of associated bacterial communities. Such categorization of TAHD lesions is critical to advance our understanding of TAHD and identify consistent microbial signatures associated with specific lesion types.

Besides potential variation in the microbial community across types of histologic lesions, it is also unknown if bacterial communities associated with TAHD differ by geographic location. First described in southwestern Washington in the early 2000s, TAHD has since been reported in other regions of the northwestern United States (4, 25). The observed prevalence of TAHD cases varies across the geographic range of TAHD, with elk herds in western Washington, western Oregon, and northwestern California being particularly affected (herein referred to as endemic TAHD areas) (4) (https://vetmed.wsu.edu/frequently-asked-questions-about-elk-hoof-disease/, https://wdfw.wa.gov/species-habitats/diseases/elk-hoof). For example, from 2016 to 2021, hunters reported hoof abnormalities in 10%–57% of elk harvested across the core endemic area of southwestern Washington (26). In contrast, TAHD cases have been identified less frequently in other areas of Washington, Oregon, and Idaho (herein referred to as sporadic TAHD areas) (4) (https://vetmed.wsu.edu/frequently-asked-questions-about-elk-hoof-disease/, https://wdfw.wa.gov/species-habitats/diseases/elk-hoof). Regional environmental conditions, such as higher rainfall and differences in land use in endemic compared to sporadic TAHD areas (27), may contribute to these geographic variations. Along with broader variation in environmental conditions, smaller-scale variation between habitats can influence the abundance and distribution of bacterial pathogens, especially when the local environment functions as a reservoir for pathogens, as in the case of some infectious hoof diseases in livestock (28–30). However, current studies of TAHD’s etiology have been limited by small sample sizes and a narrow geographic focus. Larger-scale investigations are needed to better understand the pathogens involved and how they vary across the geographic range of TAHD.

**Fig 1:**
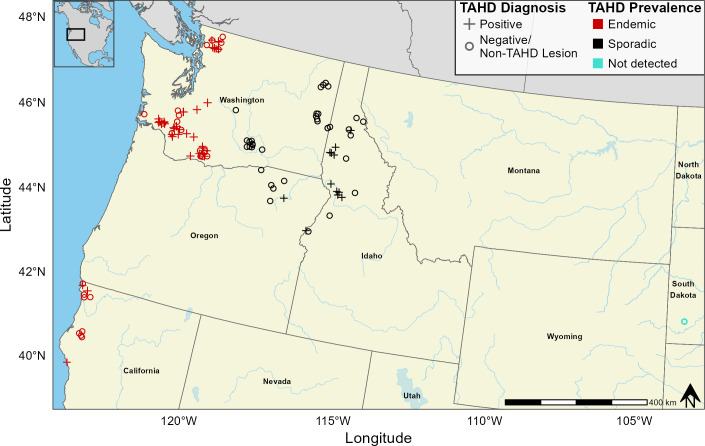
Study area. Samples originated from free-ranging elk in areas where observed occurrence of TAHD was endemic (red), sporadic (black), or not detected (teal). When coordinates were not provided with the case submission, cases were mapped based on the centroid of the reported game management unit or county. Not all cases are visible on the map because multiple cases in one area may preclude visualization. The map was created in the R programming environment using study data and open-source shapefiles.

In this study, we characterized and compared the bacterial communities in TAHD-positive lesions and normal hoof tissues from free-ranging elk from sites across the western and central United States, encompassing regions with endemic TAHD, sporadic TAHD, and no TAHD (Fig. 1). We developed a histologic categorization system for interdigital skin samples to describe the spectrum of well-defined epidermal changes within abnormal elk hooves to more accurately classify TAHD cases and non-TAHD lesions. Using this system, we identified pathogenic bacterial OTUs associated with various lesion types. Furthermore, we compared the bacterial OTUs found in TAHD-positive lesions from areas with endemic versus sporadic TAHD to explore whether regional differences in bacterial communities contribute to variation in the observed prevalence of TAHD. This research will advance our understanding of TAHD’s etiology and, ultimately, aid in the development of rapid and accurate diagnostic tests.

## MATERIALS AND METHODS

### Sample collection

We solicited hoof samples from free-ranging elk through state and federal wildlife agencies. Hooves were opportunistically collected within hours to an estimated 5 days following death from elk that were harvested, culled, or found dead between 2018 and 2021 and submitted to the Washington Animal Disease Diagnostic Laboratory (WADDL) or directly to our elk hoof disease research laboratory at Washington State University. Hoof submissions included data on collection location, age class, and sex. Samples were obtained from herds in eight collection areas within the known range of TAHD and from one area outside this range to account for potential regional variation in pathogen exposure and in environmental and host factors. Collection locations were categorized by observed regional TAHD prevalence (Fig. 1), including endemic TAHD areas (western Washington and northern California), sporadic TAHD areas (northern Idaho, central and eastern Washington, and northeastern Oregon), and an area where TAHD has not been detected (Wind Cave National Park, South Dakota).

We evaluated hooves and sampled them fresh (within days of death) or following storage at −20°C or −80°C. Hooves were rinsed with tap water to remove superficial soil and debris, assessed grossly, and photographed. Before sampling, the interdigital skin was wiped with 70% ethanol to remove superficial contamination, and approximately 100 mg of interdigital skin and subcutis was collected with sterile 4 mm punch biopsies (Integra, cat. no. 3334) for DNA extraction. In cases with grossly abnormal hooves, interdigital cutaneous ulcers were sampled, when present, or, if no ulcers were identified, the most significant interdigital gross lesion was sampled. For grossly normal hooves, a representative area of interdigital skin was sampled. Tissue samples were preserved in Allprotect (Qiagen, cat. no. 76405) and frozen at −70°C to −80°C prior to DNA extraction.

### Gross and histologic assessment

We evaluated and assigned a TAHD gross lesion grade of 0–IV based on the severity of changes (adapted from reference 2) (Table S1). Following biopsy collection, the entirety of the remaining interdigital skin and subcutis was excised and fixed in 10% neutral buffered formalin for histologic assessment. Formalin-fixed sections were processed, paraffin-embedded, sectioned, and stained by the WADDL histology laboratory according to standard protocols. Affected cases were selected for histologic review if they had at least one abnormal hoof submitted for assessment. Normal cases were selected if all four hooves appeared grossly normal (grade 0). A veterinary pathologist, blinded to gross findings, examined up to four representative full-thickness sections from each hoof, including sections adjacent to the biopsy sites. For histologically abnormal cases, sections from up to two hooves per elk were examined, while for histologically normal cases, histologic sections from two hooves were examined to confirm that the case was a suitable normal control.

**Fig 2:**
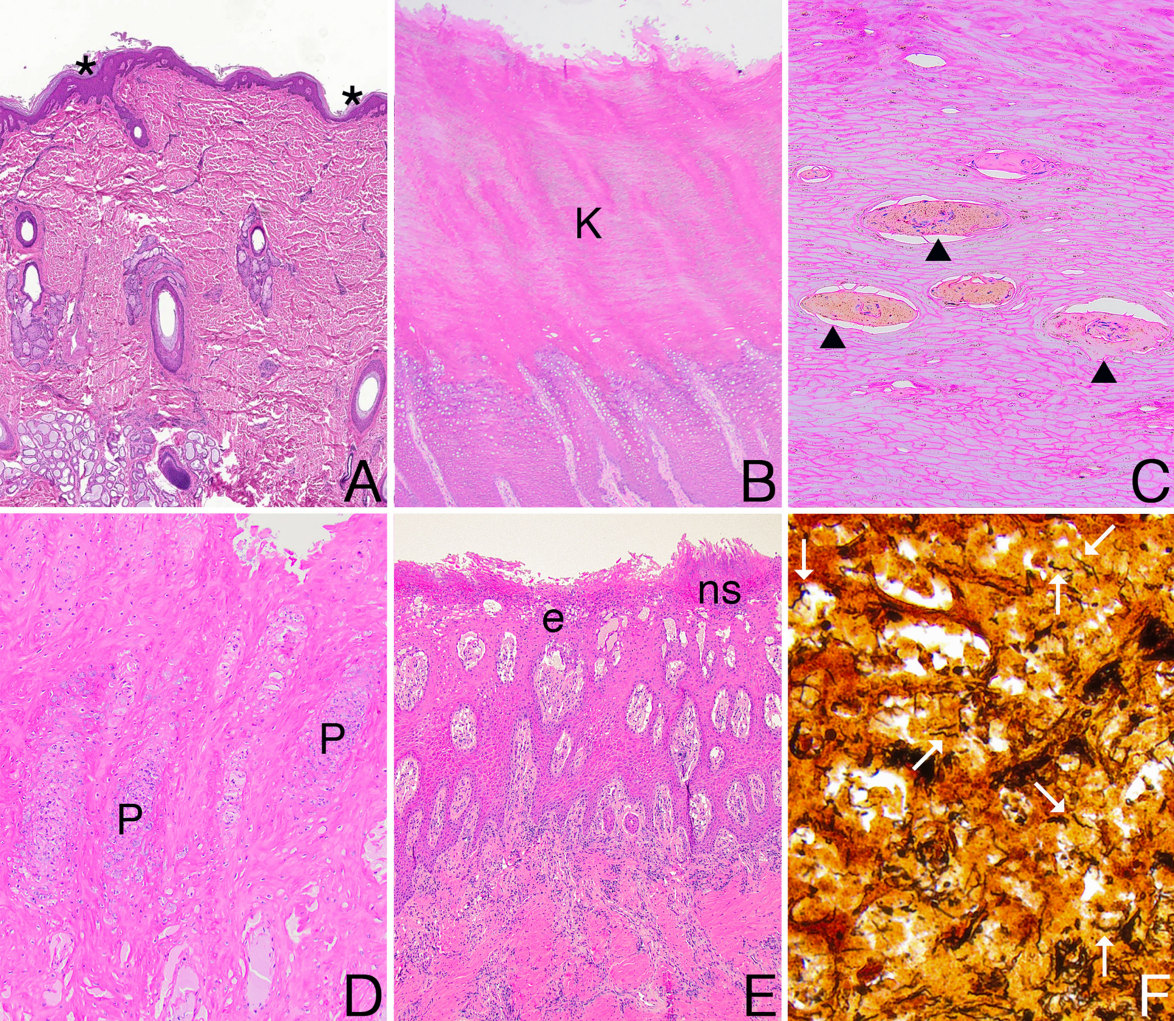
Histologic lesion categories and argyrophilic spirochetes within interdigital skin sections from elk. Representative photomicrographs of four histologic lesion categories stained with hematoxylin and eosin (**A–E**) and argyrophilic spirochetes within a TAHD lesion highlighted with a Warthin-Starry silver stain for spirochetes (**F**). All photomicrographs were white balanced to facilitate interpretation. (**A and B**) Sections of interdigital skin with normal epidermis (H_ne_) were covered with a thinner keratin layer (asterisk) in sections of haired skin above the coronary band (**A**) or a thick keratin layer (K) in sections of unhaired skin at or below the coronary band (**B**). (**C**) Lesions with intracorneal necrotic cellular debris (H_nd_) had intracorneal aggregates of necrotic cellular debris, red to brown pigment, and edema (black arrowhead). (**D**) Pustular dermatitis (H_pu_) lesions were characterized by intracorneal pustules (P). (**E**) Lesions with erosive to ulcerative dermatitis (H_eu_) included an eroded (**E**) or ulcerated epidermis subtended by bands of necrosuppurative inflammation (ns). (**F**) Numerous argyrophilic spirochetes (white arrow) within a TAHD-positive lesion.

While the identification of grossly abnormal hooves is an important first step in diagnosing TAHD, we developed a system to categorize samples by histologic abnormalities to provide the most pertinent grouping to inform further investigation of the microbiome. We described the condition of the epidermis (intact, eroded, or ulcerated) and the presence and character of any epidermal inflammation identified within histologic sections stained with hematoxylin and eosin (H&E). Following histologic assessment, we divided samples into four categories based on the abnormalities identified within H&E sections (Fig. 2). Samples with a normal epidermis (H_ne_) had normal epidermal thickness without histologically detected inflammation. Lesions characterized by intracorneal necrotic cellular debris (H_nd_) had a normal to eroded epidermis without neutrophilic inflammation. Lesions with pustular dermatitis (H_pu_) had a normal to eroded epidermis with intracorneal pustules. Finally, lesions classified as erosive to ulcerative dermatitis (H_eu_) included an eroded to ulcerated epidermis subtended by bands of neutrophilic inflammation. Each case was classified for further analyses according to the most severe histologic lesion (H_eu_ > H_pu_ > H_nd_ > H_ne_) identified within the examined sections. We expect that these categories approximate disease severity and serve as a proxy for TAHD lesion progression.

To confirm the TAHD diagnosis, sections with the most severe histologic lesion were stained with a Warthin-Starry (WS) silver stain to detect argyrophilic spirochetes. Multiple WS-stained sections were examined for cases with subtle or multiple representative lesions. TAHD-positive lesions were defined as cases with neutrophilic inflammation (H_pu_ or H_eu_ lesions) and spirochetes detected in the same hoof. All H_ne_ tissues were classified as TAHD-negative. The remaining cases that did not meet these criteria (i.e., H_nd_ lesions, H_pu_ or H_eu_ lesions without spirochetes detected) were classified as non-TAHD lesions.

To assess the correlation between gross lesions and histologic findings and to remove cases with non-representative histologic sections, we compared gross lesion grades to the most severe histologic category observed. For cases without gross grade information or cases with gross abnormalities noted that were not reflected in the examined histologic sections, we reviewed photographs from sampling. Hooves with significant gross abnormalities (i.e., extensive ulceration, significant claw overgrowth, and fractured or sloughed claws) but no neutrophilic inflammation were excluded from analysis as cases with non-representative histologic sections.

### DNA extraction and 16S rRNA gene amplicon sequencing

We thawed frozen punch biopsies at room temperature and cut them into several pieces using a sterile scalpel and forceps prior to DNA extraction. Excess normal subcutaneous adipose tissue from the deep aspect of each punch biopsy was excised and discarded when identifiable, focusing sequencing on the cutaneous tissues where TAHD lesions are detected. Tissues were placed in CKMix50-R tubes (Bertin, cat. no. P000922-LYSK0-A) and macerated using a Bertin Precellys Evolution. Next, samples were digested overnight at 55°C in a solution containing 20 μL Proteinase K, 20 μL 1 M dithiothreitol, and 180 μL solid tissue buffer (Zymo Research, cat. no. D4068). Total genomic DNA (gDNA) was extracted using the ZymoBIOMICS Miniprep Kit (Zymo Research, cat. no. D4300) following the manufacturer’s instructions. We included a negative extraction control of 75 μL of DNase/RNase-free water and a positive extraction control of macerated interdigital tissue spiked with 75 μL of a microbial community standard (Zymo Research, cat. no. D6310 or D6300) in each batch of DNA extractions. The concentration of extracted gDNA was estimated using a NanoDrop 2000c UV-Vis Spectrophotometer (Thermo Scientific, cat. no. E112352). To check for contamination, positive and negative extraction controls were confirmed via PCR amplification of the V4 region of the 16S rRNA gene using 515F-806R primers under the following conditions: 95°C for 2 min, 95°C for 30 s followed by 55°C for 1 min followed by 72°C for 2 min (repeated for 30 cycles), 72°C for 5 min, and hold at 4°C.

Extracted gDNA was submitted to the ZymoBIOMICS Targeted Sequencing Service (Zymo Research, Irvine, CA, USA) for sequencing and downstream data processing. Libraries were prepared using the Quick-16S NGS Library Prep Kit (Zymo Research, cat. no. D6400) and cleaned with the Select-a-Size DNA Clean & Concentrator Kit (Zymo Research, cat. no. D4080). Amplicon sequencing of the V3-V4 region of the 16S rRNA gene was performed on an Illumina Nextseq. Raw reads were processed using the DADA2 pipeline (31) to infer unique amplicon sequences and remove chimeras. Taxonomy was assigned using uclust (32) within QIIME v.1.9.1 (33) with a Zymo Research-curated 16S rRNA database (34). Amplicon sequence variants (ASVs) with fewer than 11 counts were removed, and OTUs from plants (class Chloroplast) and eukaryotes (family *Mitochondria*) were excluded before analysis.

### Diversity indices

Shannon diversity index (SDI), a diversity index that accounts for both taxonomic richness and evenness, was calculated using both unrarefied count data and counts rarefied to 26,051 reads, the sequencing depth of the sample with the fewest reads included in amplicon sequence analyses, with the “diversity” (or “rrarefy”) function implemented in the vegan R package (35). SDIs were compared between TAHD diagnoses (TAHD-positive versus TAHD-negative), sexes, and TAHD-positive lesions grouped by TAHD prevalence using a Welch two-sample *t*-test with the “t.test” function in R v. 4.4.1 (36). For histologic categories, age classes, extraction batches, and sequencing batches, we used analysis of variance to compare SDI with the “aov” function in R v. 4.4.1. Bray-Curtis distance matrices (measure of beta diversity) were calculated for both unrarefied and rarefied counts with the “vegdist” (or “avgdist”) function in vegan. Variation in beta diversity across metadata categories was assessed with a permutational multivariate analysis of variance using 1,000 permutations with the “adonis2” function in vegan. *P* values were adjusted for multiple comparisons using the Holm method with the “p.adjust” function in R v. 4.4.1 with significance set at *P* < 0.05. We further explored differences in beta diversity with differential abundance analysis.

### OTU-level associations with TAHD diagnosis, histologic lesion severity, and geographic prevalence

We used differential abundance analysis to identify OTUs associated with TAHD lesions and the normal skin microbiome. Differentially abundant (DA) OTUs associated with a disease are expected to be present in higher abundance in lesioned tissues when compared to normal tissues, whereas DA OTUs associated with the normal microbiome are present in higher abundance in normal tissues when compared to lesioned tissues. Because identification of bacteria at the ASV level can be inconsistent when based solely on 16S amplicon sequencing (37), we focused our differential abundance analyses on the genus level and included analyses at the family level as supplemental data. However, an important goal for this study was to identify putative pathogens of TAHD that could be targeted in future research into TAHD’s pathogenesis and during the development of confirmatory diagnostic testing to support and supplement TAHD’s current histologic diagnosis. To achieve this goal, we opted to repeat differential abundance analyses of 16S amplicon sequencing data at the ASV level. While these ASV-level identifications are putative rather than definitive, examination of 16S amplicon sequencing data at the ASV level is vital to inform future research and diagnostic test development.

We performed differential abundance analyses at the family, genus, and ASV levels with the “ancombc2” function from the ANCOMBC R package (38) to compare TAHD diagnoses (TAHD-positive lesions versus TAHD-negative tissues; *n* = 72), histologic categories (H_nd_ versus H_ne_, H_pu_ versus H_ne_, and H_eu_ versus H_ne_ of TAHD-positive lesions, TAHD-negative tissues, and non-TAHD lesions; *n* = 129), and TAHD-positive lesions by TAHD prevalence (TAHD-positive lesions from endemic versus sporadic areas; *n* = 51). This method of differential abundance analysis accounts for sample-specific and taxa-specific biases. OTUs were included in the differential abundance analyses if they were detected in at least one sample within each group included in the comparison and in ≥20% of samples. The remaining parameters were set to the default values. DA OTUs were considered significant if they had a corrected *P* value <0.05 (Holm-Bonferroni) and passed a pseudocount sensitivity test (38). To address false discovery from multiple comparisons, a pairwise directional differential abundance analysis (Dunnett’s type of test) was also performed for histologic categories, using the same parameters. Log-fold change (LFC) was calculated for DA OTUs from both analyses. We reported LFCs from the primary differential abundance analysis for comparisons between TAHD diagnoses and TAHD-positive lesions by TAHD prevalence, and LFCs from Dunnett’s type of test were reported for comparisons of histologic categories.

DA OTUs were considered associated with a group if they met four criteria: *P* < 0.05, passed the pseudocount sensitivity test, LFC > 2, and were detected in the majority of samples within a group, defined by prevalence cut points as follows. For comparisons with large sample sizes (TAHD diagnosis, *n* = 72; histologic category, *n* = 129), OTUs had to be detected in at least 50% of the samples within at least one group. For comparisons with smaller sample sizes (TAHD-positive lesions from endemic versus sporadic areas, *n* = 51), the prevalence cut point was set at 45%. For the comparison of TAHD-positive lesions by TAHD prevalence, OTUs with LFCs < −2 (i.e., associated with TAHD-positive lesions from sporadic areas) were also deemed significant. OTUs detected exclusively in diseased tissues may provide insights into disease pathogenesis. OTUs absent in at least one comparison group were considered as differentially detected (39). These OTUs were considered associated with a group if they met the respective prevalence cut point as described above.

To infer disease association, we conducted a proportion test for each OTU identified as significantly associated with a group in the TAHD-positive versus TAHD-negative and endemic versus sporadic area comparisons with the “prop.test” function in R v. 4.4.1. *P* values were adjusted for multiple comparisons using the Holm method with the “p.adjust” function in R v. 4.4.1, and adjusted *P* values <0.05 were considered significant. To further characterize associated OTUs, an odds ratio (OR) and 95% confidence interval (95% CI) were calculated for each OTU with univariate logistic regression with the “glm” function in R v. 4.4.1. Univariate ordinal logistic regression was performed with the “polr” function in the MASS R package (40) for all OTUs associated with at least one histologic category to assess the association of OTUs with histologic lesion categories. For each OTU, an OR and 95% CI were calculated. In addition to the criteria discussed above, OTUs were considered significantly associated with a histologic category if the 95% CI did not include 1, and the OTU was identified as significantly associated with either TAHD-positive lesions or TAHD-negative tissues.

Because pathogens involved in polybacterial diseases can work synergistically to initiate, prolong, or advance disease pathogenesis, we identified bacterial genera that were consistently detected concurrently within samples to identify potential keystone species with correlation analysis. We examined relative abundance data from all samples regardless of TAHD diagnosis (*n* = 129; TAHD-positive lesions, TAHD-negative tissues, and non-TAHD lesions) for the 19 genera identified as associated with TAHD-positive lesions or TAHD-negative tissues with Spearman’s rank-order correlation with the “cor” and “corpmat” functions in R v. 4.4.1. Spearman’s rank correlation coefficient (*ρ*) for all significant correlations (*P* < 0.05) was visualized with the “ggcorrplot” function in the ggcorrplot R package (41).

## RESULTS

### Sample processing and sequencing

Of the 132 samples processed for taxonomic assignment through 16S rRNA gene amplicon sequencing (Data set S1), three cases were discarded, including one case with low DNA yield (<2 ng/μL; case 202190039) and two cases with shallow sequencing depth (<26,000 reads; cases 20201976 and 202092663). Sequencing depth for 129 samples included in the final analysis ranged from 26,051 (case 202092662) to 408,560 (case 201906305) reads per sample (Fig. S1, Data set S1). All samples met the required depth for analysis as confirmed by rarefaction analysis (Fig. S2). We confirmed positive and negative extraction controls for each DNA extraction batch and did not identify any batch effects associated with extraction or sequencing batches (Data set S2).

### Histologic and gross lesions

Histologic lesions (H_nd_, H_pu_, or H_eu_) were identified in 108 cases (Table 1, Fig. 2). Overall, 57% of cases (74/129; Table 1, Fig. 2) contained neutrophilic inflammation (H_pu_ or H_eu_). Spirochetes were detected in 69% of cases with neutrophilic inflammation (51/74; Table 1, Fig. 2), resulting in classification as TAHD-positive lesions. When TAHD-positive lesions from endemic and sporadic areas were examined separately, 38% of TAHD-positive lesions from endemic areas (15/39; Table 1) and 50% of those from sporadic areas (6/12) had H_pu_ lesions, whereas H_eu_ lesions were identified in 62% of TAHD-positive lesions from endemic areas (24/39) and 50% of those from sporadic areas (6/12). *Spirochaetaceae* signatures were detected by 16S amplicon sequencing in 30% of non-TAHD lesions with neutrophilic inflammation (H_pu_ or H_eu_; 7/23; Data set S1).

**TABLE 1:** Case characteristics^*a*^

Case characteristics	Histologic category								Total (*n* = 129)	
	Normal epidermis (H_ne_) (*n* = 21)		Intracorneal necrotic cellular debris (H_nd_) (*n* = 34)		Pustular dermatitis (H_pu_) (*n* = 37)		Erosive to ulcerative dermatitis (H_eu_) (*n* = 37)			
TAHD diagnosis										
Positive	0	(0%)	0	(0%)	21	(57%)	30	(81%)	51	(40%)
Negative	21	(100%)	0	(0%)	0	(0%)	0	(0%)	21	(16%)
Non-TAHD lesions	0	(0%)	34	(100%)	16	(43%)	7	(19%)	57	(44%)
TAHD prevalence										
Endemic	6	(29%)	8	(24%)	24	(65%)	28	(76%)	66	(51%)
TAHD-positive only					15	(38%)	24	(62%)	39	
Sporadic	5	(24%)	22	(65%)	11	(30%)	9	(24%)	47	(36%)
TAHD-positive only					6	(50%)	6	(50%)	12	
Not detected	10	(48%)	4	(12%)	2	(5%)	0	(0%)	16	(12%)
Sex										
Male	8	(38%)	11	(32%)	17	(46%)	22	(59%)	58	(45%)
Female	13	(62%)	23	(68%)	20	(54%)	15	(41%)	71	(55%)
Age class										
Adult	19	(90%)	32	(94%)	36	(97%)	32	(86%)	119	(92%)
Calf	2	(10%)	2	(6%)	1	(3%)	3	(8%)	8	(6%)
Unknown	0	(0%)	0	(0%)	0	(0%)	2	(5%)	2	(2%)

^*a*^ Descriptive data from elk sampled for histologic assessment of interdigital skin and 16S rRNA gene amplicon sequencing for microbiome analysis.

Gross lesion grades were available for 90% of cases regardless of TAHD diagnosis (116/129; Table S1) and for 94% of cases with TAHD-positive lesions (48/51; Table S2). Of these, 92% of cases with TAHD-positive lesions (44/48; Table S2) had gross hoof abnormalities identified during sampling (grades I–IV), with grade IV lesions most frequently identified (28/48, 58%). When TAHD-positive lesions were broken down by TAHD prevalence, 75% (27/36; Table S2) of TAHD-positive cases from endemic areas exhibited grade IV lesions compared to 8% (1/12) of TAHD-positive cases from sporadic areas. No gross hoof abnormalities (grade 0) were detected in the remaining 8% of cases with TAHD-positive lesions (4/48; Table S2). Of the cases diagnosed as TAHD-negative tissues (*n* = 21; Table 1), 19% (4/21; Table S2) had grade I or grade II gross lesions described during sampling. Of the lesions classified as H_nd_ (*n* = 34; Table 1), a histologic lesion presumed to be early inflammation between H_ne_ tissues and lesions with neutrophils (H_pu_ and H_eu_), 71% of cases with gross grades available (20/28; Table S1) were identified in grossly normal hooves (grade 0) while the remainder were in hooves classified as gross grades I–III (8/28, 29%).

### Diversity indices

**Fig 3:**
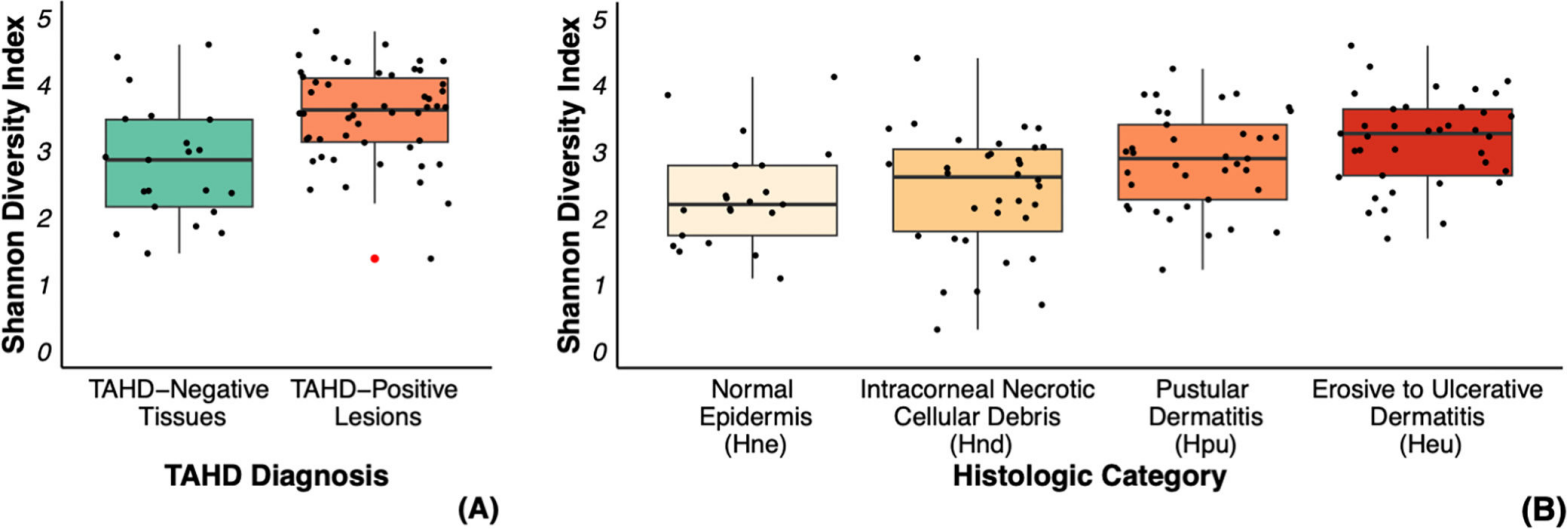
SDI, a measure of alpha diversity, differed significantly by TAHD diagnosis in elk (**A**) and histologic category (**B**). Scatterplots by category with overlay of summary boxplots (median, 25th quartile, and 75th quartile), whiskers (1.5× interquartile range, IQR), and outliers (red dots, >1.5× IQR).

TAHD-positive lesions had a significantly higher SDI compared to TAHD-negative tissues (*P* < 0.05; Fig. 3A), and SDI increased as histologic lesion severity progressed from H_ne_ to H_nd_ to H_pu_ to H_eu_ (*P* < 0.05; Fig. 3B). No significant differences in SDI were observed between sexes, age classes, or TAHD-positive lesions from endemic versus sporadic areas (Data set S2). Bray-Curtis dissimilarity indices differed significantly between TAHD-positive lesions and TAHD-negative tissues, histologic categories, TAHD-positive cases from endemic versus sporadic areas, and sexes, but did not differ between age classes (Data set S2).

### Bacterial OTUs associated with TAHD-positive lesions

**Fig 4:**
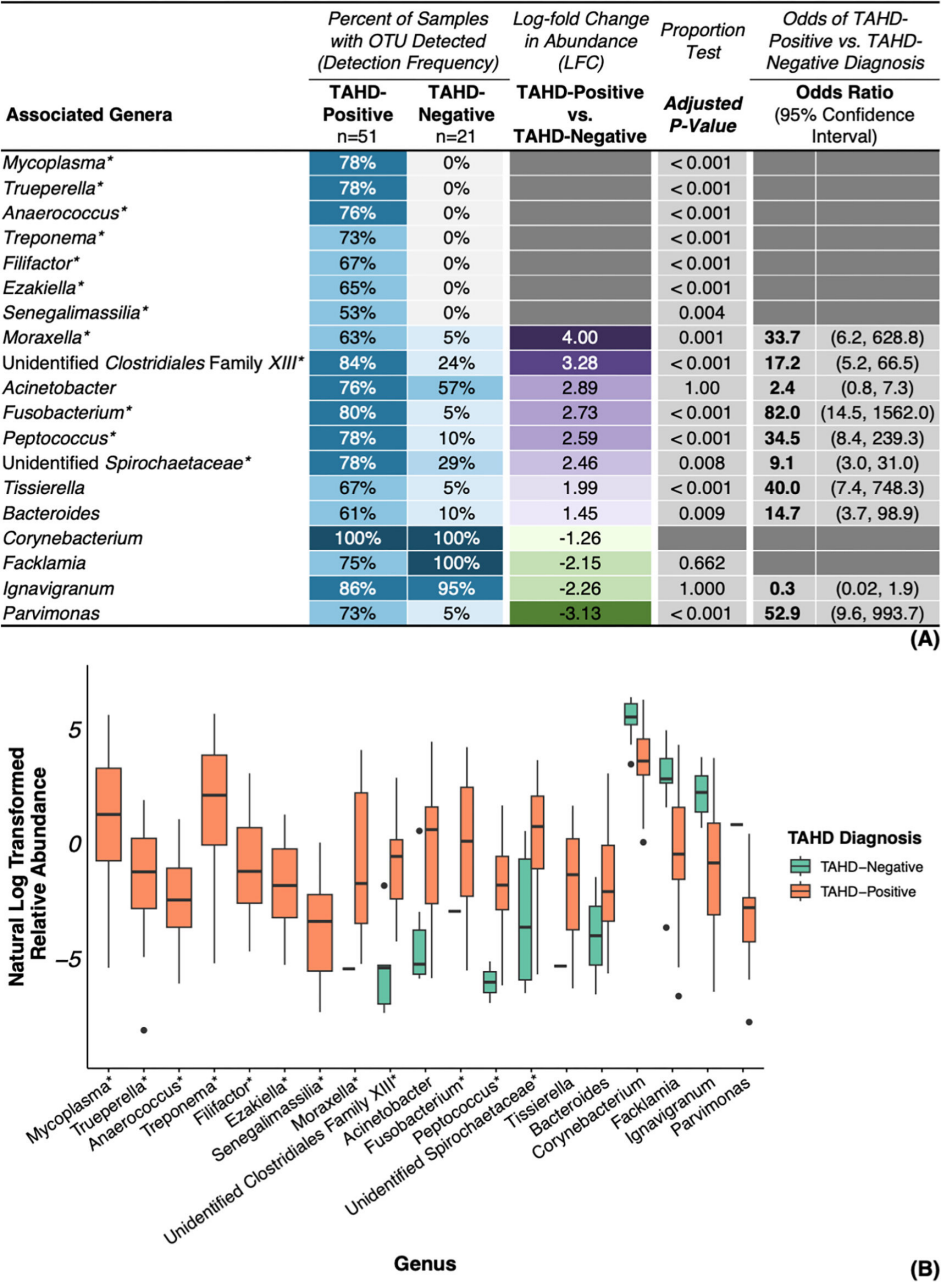
Genera associated with TAHD-positive lesions or TAHD-negative tissues. (**A**) Heatmap of detection frequencies for genera associated with TAHD-positive lesions or TAHD-negative tissues with additional significance criteria. (**B**) Boxplot summarizing natural log transformed relative abundance for associated genera by TAHD diagnosis with boxplots (median, 25th quartile, and 75th quartile), whiskers (1.5× IQR), and outliers (black dots, >1.5× IQR). *, genus met all established significance criteria.

**Fig 5:**
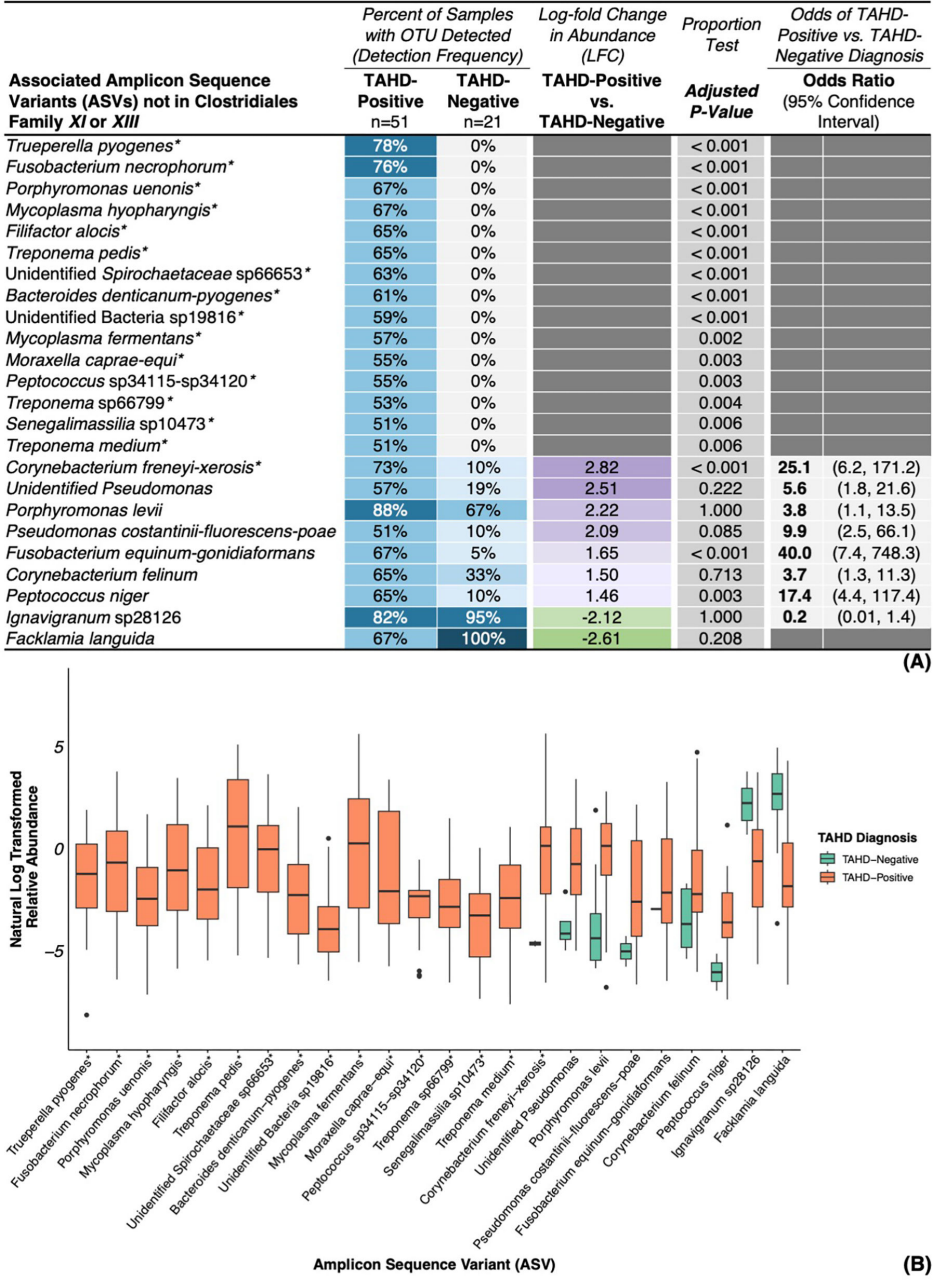
ASVs not in Clostridiales Family *XI* or *XIII* associated with TAHD-positive lesions or TAHD-negative tissues. (**A**) Heatmap of detection frequencies for ASVs associated with TAHD-positive lesions or TAHD-negative tissues with additional significance criteria. (**B**) Boxplot summarizing natural log transformed relative abundance for associated ASVs by TAHD diagnosis with boxplots (median, 25th quartile, and 75th quartile), whiskers (1.5× IQR), and outliers (black dots, >1.5× IQR). *, ASV met all established significance criteria.

We identified several bacterial families (Fig. S3), genera (Fig. 4), and ASVs (Fig. 5 and Fig. S4) that were significantly associated with TAHD-positive lesions. OTUs were considered associated with TAHD-positive lesions if they met the following criteria: detection in at least 50% of TAHD-positive lesions, a significant difference in detection frequency between TAHD-positive lesions and TAHD-negative tissues (*P* < 0.05), and an LFC > 2.

**Fig 6:**
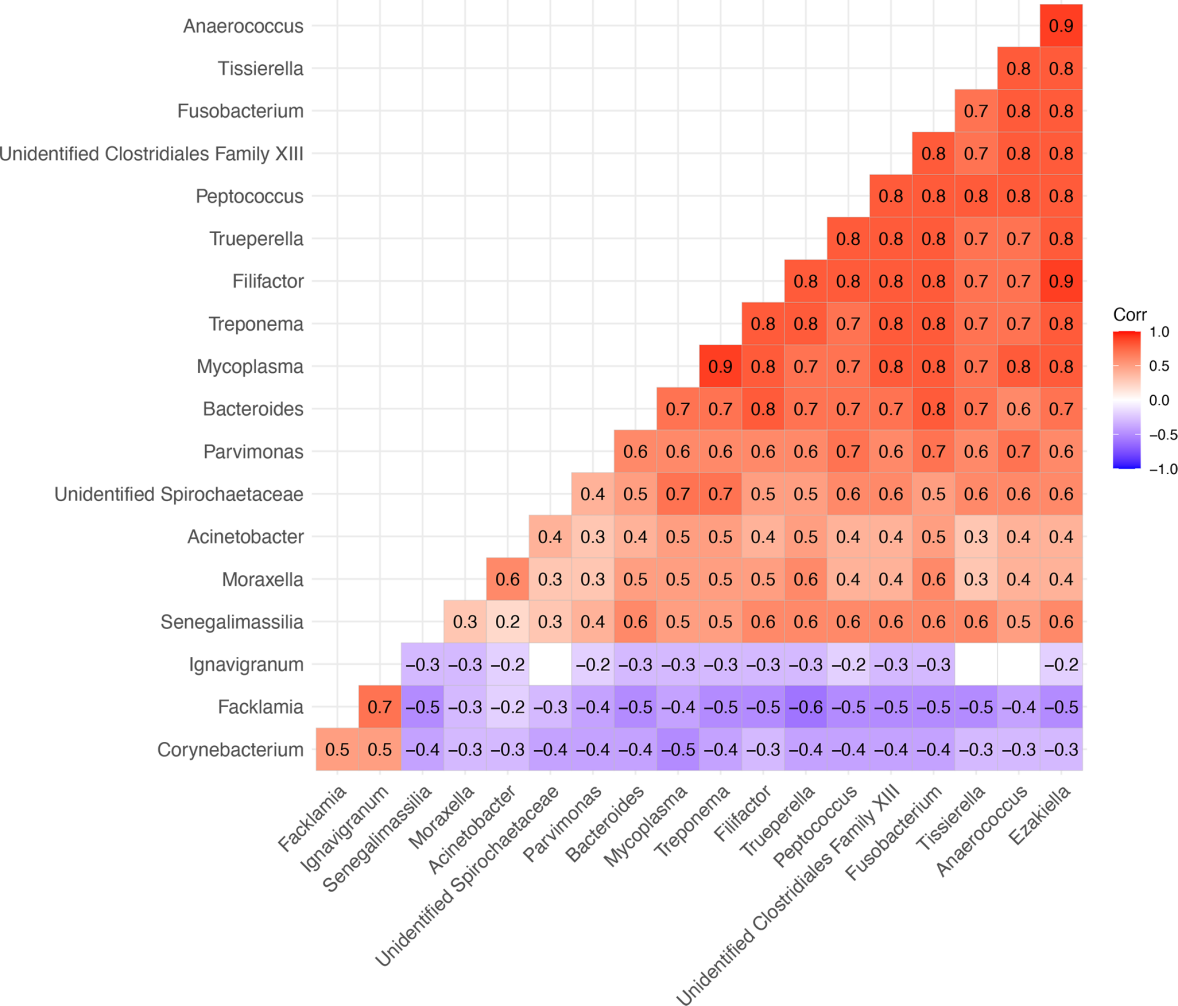
Matrix of Spearman’s rank correlation coefficients for genera associated with TAHD-positive lesions or TAHD-negative tissues. Pairwise correlation matrix of 19 genera identified as associated with TAHD-positive lesions or TAHD-negative tissues. Tile colors reflect value of Spearman’s rank correlation coefficient (*ρ*). Tiles only included for significant correlations (*P* < 0.05).

Among the most notable findings within the family *Spirochaetaceae*, *Treponema* and unidentified *Spirochaetaceae* were detected at high frequencies (73% and 78%, respectively; Fig. 4) with notable enrichment in TAHD-positive lesions (unidentified *Spirochaetaceae*, LFC = 2.46, OR = 9.1, 95% CI = 3.0, 31.0). In contrast, these genera were either absent (*Treponema*, *P* < 0.001; Fig. 4) or infrequently detected in TAHD-negative tissues (unidentified *Spirochaetaceae*, 29%, *P* = 0.008), indicating that both genera were strongly associated with TAHD. *Treponema* also had the highest median relative abundance of all genera associated with TAHD-positive lesions (Fig. 4B). Similarly, *Mycoplasma* and its family (*Mycoplasmataceae*) were detected in 78% of TAHD-positive lesions but were absent from all TAHD-negative tissues (*P* < 0.001; Fig. 4 and Fig. S3). Correlation analysis identified a remarkably strong positive correlation between *Treponema* and *Mycoplasma* (*ρ* = 0.9, *P* < 0.05) as well as strong positive correlations between both genera and unidentified *Spirochaetaceae* (*ρ* = 0.7, *P* < 0.05) along with nine other genera (*ρ* = 0.7–0.8, *P* < 0.05; Fig. 6). At the ASV level, members of *Spirochaetaceae* (*T. pedis*, unidentified *Spirochaetaceae* sp66653, *Treponema* sp66799, and *T. medium*) and *Mycoplasmataceae* (*M. hyopharyngis* and *M. fermentans*) were associated with TAHD-positive lesions (51%–67%, *P* ≤ 0.006; Fig. 5) and were not detected in any TAHD-negative tissues.

Several other genera and ASVs were also detected exclusively in TAHD-positive lesions. These genera included *Trueperella* (*Actinomycetaceae*), *Filifactor* (*Peptostreptococcaceae*), *Senegalimassilia* (*Coriobacteriaceae*), *Anaerococcus* (Clostridiales Family *XI*), and *Ezakiella* (Clostridiales Family *XI*) with detection frequencies ranging from 53% to 78% of TAHD-positive lesions (*P* ≤ 0.004; Fig. 4). ASVs related to these genera that were also associated with TAHD-positive lesions included *Trueperella pyogenes*, *Filifactor alocis*, *Senegalimassilia* sp10473, and nine known or poorly characterized ASVs within Clostridiales Family *XI* (51%–82%, *P* ≤ 0.006; Fig. 5 and Fig. S4). ASVs from other genera, such as *Porphyromonas uenonis*, *Bacteroides denticanum-pyogenes*, *and* unidentified Bacteria sp19816, were also detected in most TAHD-positive lesions (59%–67%, *P* < 0.001; Fig. 5) without any detections in TAHD-negative tissues.

Similar to the genera *Treponema* and *Mycoplasma*, the genus *Fusobacterium* (*Fusobacteriaceae*) was also significantly enriched in TAHD-positive lesions (LFC = 2.73; Fig. 4), with frequent identification in TAHD-positive lesions (80%, *P* < 0.001) relative to rare detection in TAHD-negative tissues (5%). Interestingly, *Fusobacterium* exhibited the highest odds ratio of all differentially abundant genera associated with TAHD-positive lesions (OR = 82.0, 95% CI = 14.5, 1,562.0; Fig. 4). Other genera, including *Peptococcus* (*Peptococcaceae*), *Moraxella* (*Moraxellaceae*), and unidentified Clostridiales Family *XIII* (Clostridiales Family *XIII*) were also significantly enriched in TAHD-positive lesions (LFC = 2.59–4.00, OR = 17.2–34.5; Fig. 4) with detection frequencies in TAHD-positive lesions ranging from 63% to 84% (*P* ≤ 0.001). In contrast, these genera were infrequently detected at lower relative abundances in TAHD-negative tissues (5%–24%; Fig. 4). Of these, both *Fusobacterium* and *Peptococcus* were strongly and positively correlated with each other, *Treponema*, *Mycoplasma*, and eight other genera (*ρ* = 0.7–0.8, *P* < 0.05; Fig. 6).

ASVs related to the differentially abundant genera discussed above, including *Fusobacterium necrophorum*, *Moraxella caprae-equi*, *Peptococcus* sp34115-sp34120, and unidentified Clostridiales Family *XIII* sp31685 and sp31686, were detected in most TAHD-positive lesions (55%–80%, *P* ≤ 0.003; Fig. 5 and Fig. S4) but were absent from all TAHD-negative tissues. While the genus *Corynebacterium* and its family (*Corynebacteriaceae*) were detected in all samples included in the study with enrichment in TAHD-negative tissues (Fig. 4 and Fig. S3), a single related ASV, *Corynebacterium freneyi-xerosis*, was significantly enriched in TAHD-positive lesions (LFC = 2.82, OR = 25.1, 95% CI = 6.2, 171.2, *P* < 0.001; Fig. 5) with increased detection frequency in TAHD-positive lesions (73%) relative to TAHD-negative tissues (10%).

### Bacterial trends across histologic lesion severity (H_ne_ → H_nd_ → H_pu_ → H_eu_)

**Fig 7:**
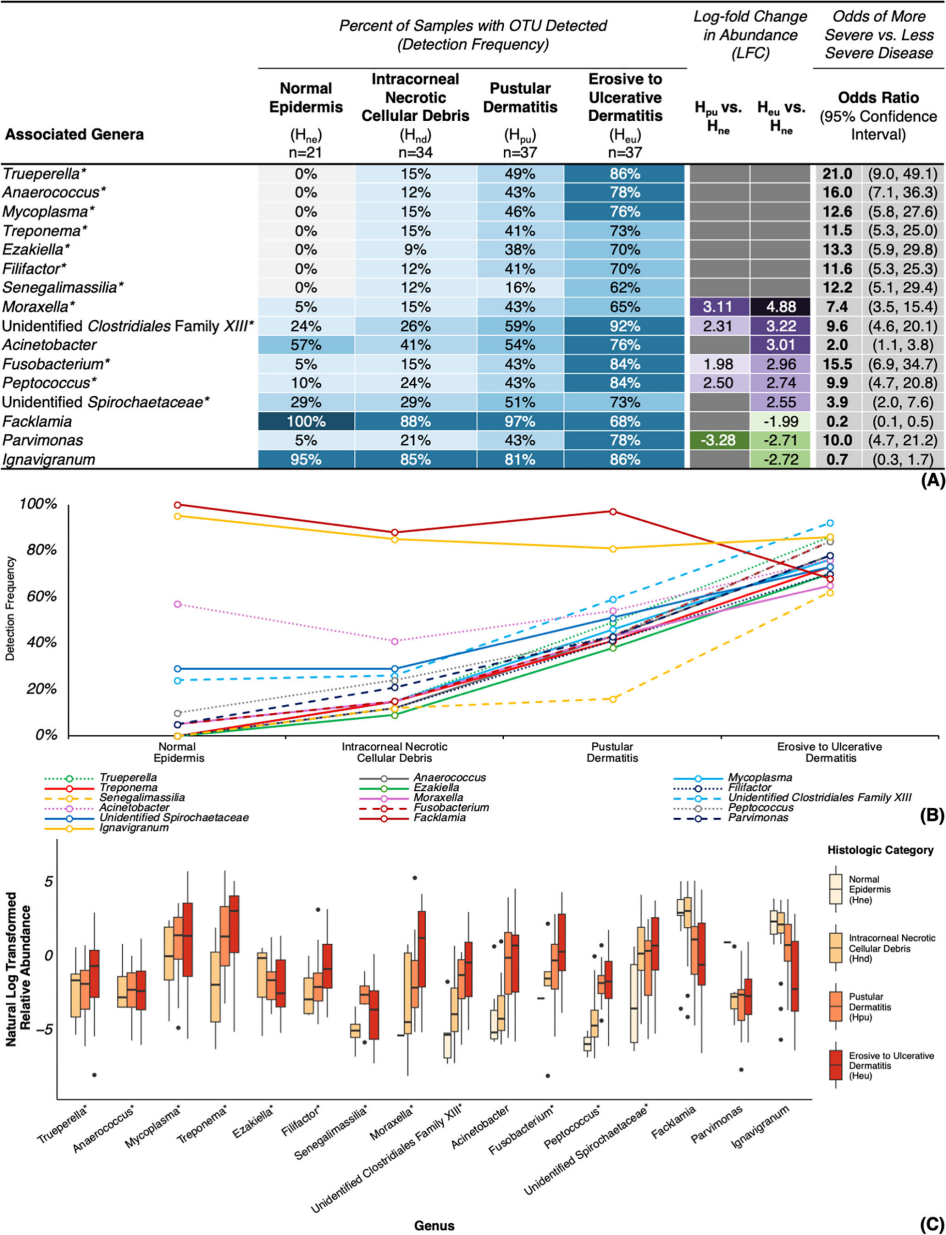
Genera associated with histologic lesion categories. (**A**) Heatmap of detection frequencies of all samples (TAHD-positive, TAHD-negative, and non-TAHD lesions) for genera associated with both histologic lesion categories and TAHD diagnoses (TAHD-positive lesions or TAHD-negative tissues) with additional significance criteria. (**B**) Line graph of detection frequencies for associated genera by histologic category. (**C**) Boxplot summarizing natural log transformed relative abundance for associated genera by histologic category with boxplots (median, 25th quartile, and 75th quartile), whiskers (1.5× IQR), and outliers (black dots, >1.5× IQR). *, genus met all established significance criteria.

**Fig 8:**
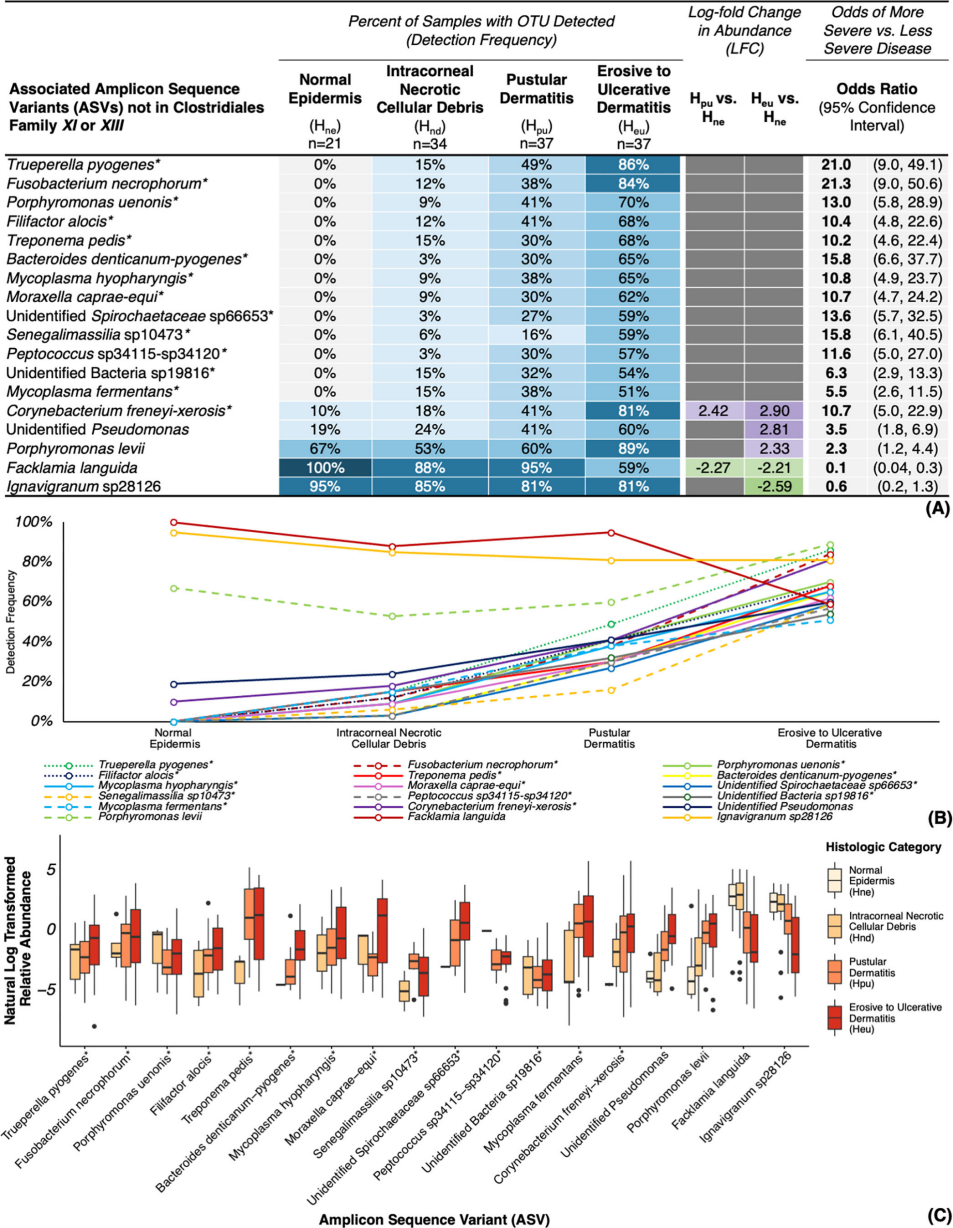
ASVs not in Clostridiales Family *XI* or *XIII* associated with histologic lesion categories. (**A**) Heatmap of detection frequencies of all samples (TAHD-positive, TAHD-negative, and non-TAHD lesions) for ASVs not in Clostridiales Family *XI* or *XIII* associated with both histologic lesion categories and TAHD diagnoses (TAHD-positive lesions or TAHD-negative tissues) with additional significance criteria. (**B**) Line graph of detection frequencies for associated ASVs by histologic lesion category. (**C**) Boxplot summarizing natural log transformed relative abundance for associated ASVs by histologic category with boxplots (median, 25th quartile, and 75th quartile), whiskers (1.5× IQR), and outliers (black dots, >1.5× IQR). *, ASV met all established significance criteria.

Comparison of histologic lesion categories across all samples (TAHD-positive lesions, TAHD-negative tissues, and non-TAHD lesions) revealed that most families (Fig. S5), genera (Fig. 7), and ASVs (Fig. 8 and Fig. S6) associated with TAHD-positive lesions also exhibited significant and progressive increases in detection frequency and abundance as lesion severity advanced based on the significance criteria described previously. For example, genera *Treponema*, *Mycoplasma*, *Trueperella*, *Filifactor*, *Anaerococcus*, and *Senegalimassilia* were not detected in any H_ne_ tissues but were associated with more severe histologic lesions (OR = 11.5–21.0) with increasing detection frequency as lesion severity progressed from H_nd_ to H_eu_ (Fig. 7) and detection at lower relative abundances in H_nd_ lesions relative to more severe histologic lesions (Fig. 7C). *Ezakiella* was absent from H_ne_ tissues and associated with more severe histologic lesions (OR = 13.3, 95% CI = 5.9, 29.8) with increased detection frequency as lesion severity progressed (Fig. 7); however, the genus did not exhibit a corresponding increase in relative abundance with progression of lesion severity (Fig. 7C). While infrequently detected in H_ne_ tissues (5%–24%), *Fusobacterium*, *Peptococcus*, *Moraxella*, and unidentified Clostridiales Family *XIII* were associated with more severe histologic lesions (OR = 7.4–15.5) and were detected with increased frequency (Fig. 7) and abundance in both H_pu_ (43%–59%, LFC = 1.98–3.11) and H_eu_ lesions (65%–92%, LFC = 2.74–4.88). Unidentified *Spirochaetaceae* were associated with more severe histologic lesions (OR = 3.9, 95% CI = 2.0, 7.6) with more frequent detection at significantly higher abundance in H_eu_ lesions (73%, LFC = 2.55; Fig. 7) relative to H_ne_ tissues (29%).

At the ASV level, members of *Spirochaetaceae* (*T. pedis* and unidentified *Spirochaetaceae* sp66653), *Mycoplasmataceae* (*M. hyopharyngis* and *M. fermentans*), and *Fusobacteriaceae* (*F. necrophorum*) showed a steady increase in detection frequency as histologic lesion severity advanced from H_nd_ to H_eu_ (OR = 5.5–21.3; Fig. 8) with no detections in H_ne_ tissues. *T. pyogenes*, *P. uenonis*, *F. alocis*, *B. denticanum-pyogenes*, *M. caprae-equi*, *S*. sp10473, unidentified Bacteria sp19816, *P*. sp34115-sp34120, and 11 known or poorly characterized ASVs within Clostridiales Families *XI* and *XIII* were associated with more severe histologic lesions (OR = 6.3–33.2; Fig. 8 and Fig. S6), with more frequent detection in severe histologic lesions and no detections in H_ne_ tissues. Of the ASVs associated with TAHD-positive lesions, *C. freneyi-xerosis* was the only ASV detected in H_ne_ tissues (Fig. 8) that was also associated with more severe histologic lesions (OR = 10.7, 95% CI = 5.0, 22.9). We also identified significant enrichment and increased detection frequency of *C. freneyi-xerosis* in both H_pu_ (41%, LFC = 2.42; Fig. 8) and H_eu_ lesions (81%, LFC = 2.90) relative to H_ne_ tissues (10%).

### Comparison of bacterial communities in TAHD-positive lesions from endemic and sporadic areas

**Fig 9:**
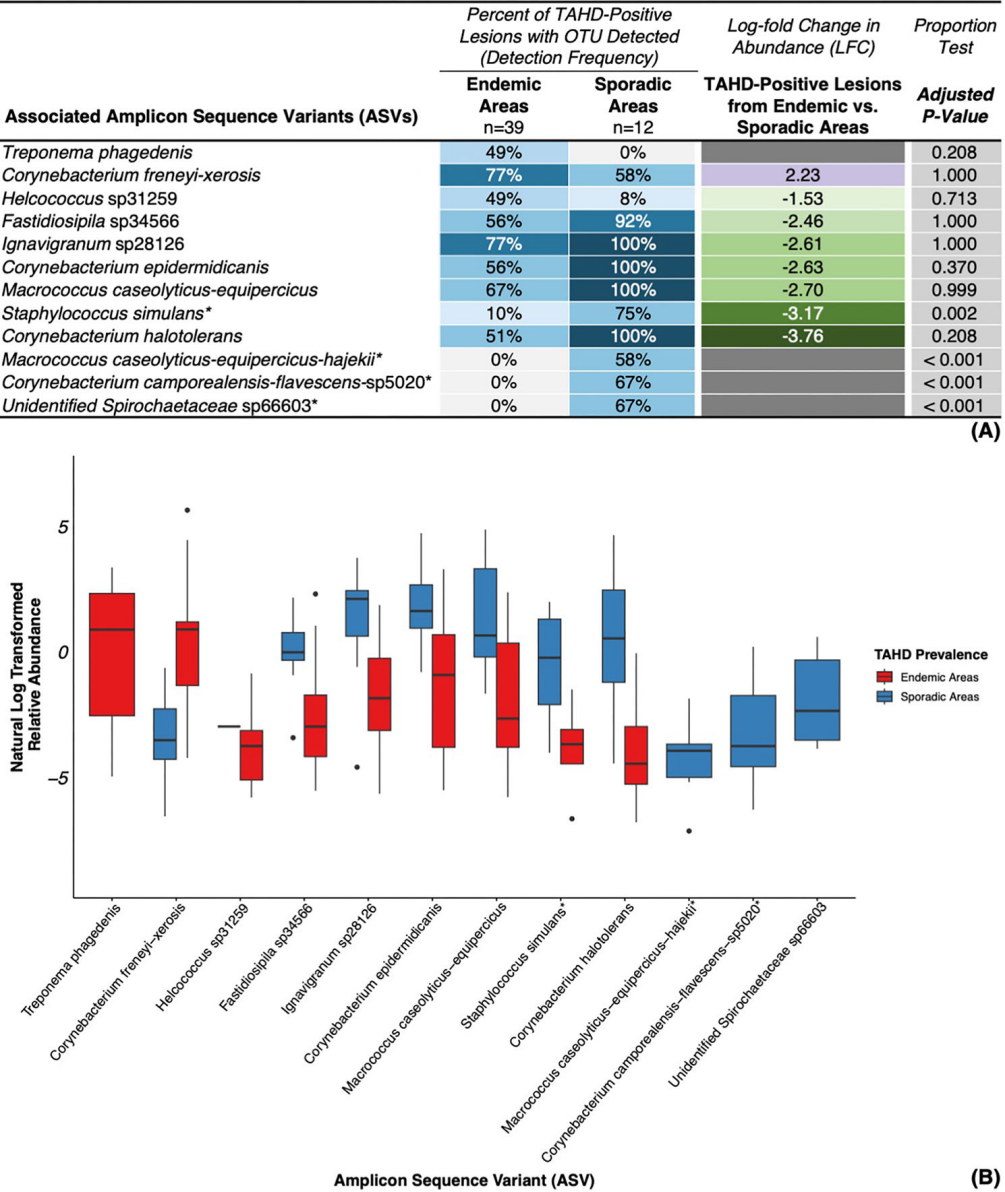
ASVs associated with TAHD-positive lesions from endemic versus sporadic areas. (**A**) Heatmap of detection frequencies for ASVs associated with TAHD-positive lesions from endemic or sporadic areas with additional significance criteria. (**B**) Boxplot summarizing natural log transformed relative abundance for associated ASVs for TAHD-positive lesions from endemic or sporadic areas with boxplots (median, 25th quartile, and 75th quartile), whiskers (1.5× IQR), and outliers (black dots, >1.5× IQR). *, ASV met all established significance criteria.

Comparison of TAHD-positive lesions from endemic (*n* = 39) and sporadic (*n* = 12) areas revealed minimal structural differences in bacterial community composition across taxonomic levels (Fig. 9; Fig. S7 and S8) based on significance criteria described previously. Unidentified *Spirochaetaceae* sp66603, a member of both a family and a genus-level group that were associated with TAHD-positive lesions in this study (Fig. 4 and Fig. S3), was not detected in endemic areas but was detected in 67% of TAHD-positive lesions from sporadic areas (8/12, *P* < 0.001; Fig. 9). Further investigation of this ASV identified it in normal tissues from areas where TAHD has not been detected (Data set S1). The ASV *T. phagedenis* was detected in 49% of TAHD-positive lesions from endemic areas (19/39; Fig. 9) with no detections in TAHD-positive lesions from sporadic areas, although these differences were not statistically significant (*P* = 0.208). Although the ASV *C. freneyi-xerosis* was detected with similar frequency in TAHD-positive lesions from endemic (77%, *P* = 1.000; Fig. 9) and sporadic (58%) areas, this ASV was significantly enriched in TAHD-positive lesions from endemic areas (LFC = 2.23). Other ASVs, including *Staphylococcus simulans*, *Macrococcus caseolyticus-equipercicus-hajekii*, and *Corynebacterium camporealensis-flavescens*-sp5020, were also associated with TAHD-positive lesions from sporadic areas (58%–75%, *P* ≤ 0.002; Fig. 9) with rare (10%, *S. simulans*) or no detections (*M. caseolyticus-equipercicus-hajekii* and *C. camporealensis-flavescens-*sp5020) in TAHD-positive lesions from endemic areas.

## DISCUSSION

Microbial community analysis of 16S rRNA gene amplicon sequencing data revealed that TAHD-positive lesions had higher microbial diversity, assessed with the SDI, compared to TAHD-negative tissues (Fig. 3A), with an increase in diversity as lesion severity progressed (Fig. 3B). This finding is in contrast to the lower alpha diversity that is typically described in other polymicrobial diseases like BDD and CODD (18, 42–44). Overall, however, these findings are consistent with the findings of a previous study of TAHD (4) and suggest that TAHD lesions are characterized by distinct shifts in microbial communities with enrichment of microbial signatures associated with progression of lesion severity.

Histologic detection of spirochetes within TAHD lesions is currently the cornerstone of TAHD’s case definition. In addition to histologic identification of argyrophilic spirochetes, we confirmed detection and significant enrichment of *Spirochaetaceae* and *Treponema* in TAHD-positive lesions, reaffirming their association with TAHD lesions. Consistent with previous reports (3, 4, 6, 22), we confirmed the association of the genera *Treponema* and unidentified *Spirochaetaceae* with TAHD-positive lesions (Fig. 4). *Treponema* was exclusively detected in TAHD-positive lesions (Fig. 4) with detection frequencies and relative abundances that steadily increased as lesions progressed from intracorneal necrotic cellular debris (H_nd_) to erosive to ulcerative dermatitis (H_eu_; Fig. 7). Four spirochete ASVs, *T. pedis*, unidentified *Spirochaetaceae* sp66653, *T*. sp66799, and *T. medium*, were detected in the majority of TAHD-positive lesions and were absent from TAHD-negative/normal (H_ne_) tissues (Fig. 5 and 8). Some of these ASVs may represent novel or poorly characterized *Spirochaetaceae* variants (15, 22). Notably, *T. pedis* and sp66653 were absent in TAHD-negative/H_ne_ tissues (Fig. 8), and their detection frequency and relative abundance increased as lesion severity progressed from H_nd_ to H_eu_, indicating a strong association with TAHD and that these specific bacterial ASVs may have greater clinical relevance. However, because unidentified *Spirochaetaceae* were also detected in 29% of TAHD-negative tissues (ASVs sp66598 and sp66603; Data set S1), it remains undetermined if this group includes commensal or less pathogenic ASVs.

While regional differences in the detection of certain spirochetes in TAHD lesions between endemic and sporadic areas were observed, the relevance of this finding remains unclear. Unidentified *Spirochaetaceae* sp66603 was detected in TAHD-positive lesions from areas with sporadic, but not endemic, TAHD; however, this ASV was also detected in non-TAHD lesions from sporadic areas and in both TAHD-negative/H_ne_ tissues and non-TAHD lesions from areas with no TAHD detections (Fig. 9; Data set S1). It is currently unknown if unidentified *Spirochaetaceae* sp66603 is less pathogenic than other spirochetes or if, when present with other bacteria associated with TAHD, it may occupy a niche within TAHD lesions that more virulent spirochetes fill in endemic areas. Although in this study, *T. phagedenis* was exclusively detected in TAHD lesions from endemic areas, it has been previously reported in TAHD-positive lesions in sporadic areas (4). Collectively, these findings support the idea that TAHD’s etiology includes multiple spirochetes. Associated spirochetes may produce virulence factors, such as major sheath protein, that facilitate bacterial persistence in diseased tissues and modulate the host immune response (7–9, 45). They also may form synergistic biofilms with other bacteria that promote invasion of host tissues and the proliferation of select bacterial species (46).

The genus *Mycoplasma*, along with ASVs *M. hyopharyngis* and *M. fermentans*, was detected exclusively in TAHD-positive lesions (Fig. 4 and 5), and, similar to treponemes, we detected *Mycoplasma*, *M. hyopharyngis*, and *M. fermentans* with increasing frequency as histologic lesion severity progressed from H_nd_ to H_eu_ (Fig. 7 and 8). Furthermore, the detection and abundance of *Mycoplasma* were strongly and positively correlated with *Treponema* (Fig. 6) with a remarkably high correlation coefficient (*ρ* = 0.9), suggesting that these genera may co-occur within TAHD lesions. Mycoplasmas cannot be visualized with routine histologic stains but have been consistently detected in digital dermatitis lesions in livestock and TAHD lesions in elk using 16S rRNA gene amplicon sequencing (4, 10, 18, 47). These results suggest that TAHD is likely a polybacterial disease where *Mycoplasma* plays an important synergistic role. Mycoplasmas are uniquely adapted to play a central role in polymicrobial infections by forming biofilms and establishing intracellular infections within epithelial cells to support their parasitic lifestyle within a host (48, 49). Mycoplasmas also employ multiple strategies to evade the host immune system through antigenic variation and direct interference with the host’s antibodies (50–53).

Although not previously reported in digital dermatitis or TAHD, we found that *Corynebacterium freneyi-xerosis* was associated with TAHD-positive lesions across its geographic range (Fig. 5). *C. freneyi-xerosis* was detected at higher abundance in the majority of TAHD-positive lesions (73%, LFC = 2.82; Fig. 5) with identification at lower abundance in a few TAHD-negative/H_ne_ tissues (10%), indicating a strong association with TAHD. We also identified increased detection frequency and relative abundance of *C. freneyi-xerosis* as lesion severity progressed from H_ne_ tissues to H_eu_ lesions (Fig. 8), further supporting its association with pododermatitis in elk. *C. freneyi-xerosis* was also detected in higher abundance and with greater frequency in TAHD-positive lesions from endemic areas relative to those from sporadic areas (Fig. 9), suggesting that it may be associated with the higher prevalence of TAHD observed in endemic TAHD areas or with more chronic TAHD lesions represented by hooves with gross grade IV lesions (Table S2). While ASV-level identifications should be interpreted with caution and considered as putative species-level identifications pending confirmation with full 16S gene sequencing, construction of metagenome-assembled genomes, or isolation with bacterial culture, our findings underscore the importance of considering microbiome data at the ASV level, especially for families and genera dominated by normal microbiota (OTUs with an LFC < 0 or OR < 1), such as the genera *Corynebacterium*, *Facklamia*, and *Ignavigranum* (Fig. 4 and 7) and families *Corynebacteriaceae* and *Aerococcaceae* (Fig. S3 and S5). In the case of *C. freneyi-xerosis*, both bacterial species represented by this ASV, *C. freneyi* and *C. xerosis*, have been previously linked to clinically significant infections in other species (54–56), and several other *Corynebacteri*a have been implicated in well-characterized, keratinolytic, erosive, skin diseases in humans that are often diagnosed in feet exposed to moist and warm conditions (57–59). A wet environment and muddy pasture conditions have also been implicated as risk factors for hoof disease in livestock associated with BDD and CODD (60–62). Increased environmental moisture in endemic areas (27) may contribute to the higher abundance and frequency of *C. freneyi-xerosis* and other TAHD-related bacteria in TAHD-positive lesions from these areas. Moreover, a prospective study of BDD lesions in dairy cattle identified a group of early *Treponema*-independent lesions associated with genus *Corynebacterium*, among other OTUs, that preceded more typical digital dermatitis pathogens (10).

We also identified a group of OTUs of interest to the etiology of early TAHD lesions, ASV *Macrococcus caseolyticus-equipercicus*, with its genus and family. These OTUs were the only OTUs identified in 100% of H_nd_ lesions (Data set S1). In our comparison of TAHD-positive lesions from endemic and sporadic areas, *M. caseolyticus-equipercicus* and a closely related ASV, *M. caseolyticus-equipercicus-hajekii*, were also enriched in or associated with TAHD-positive lesions from sporadic TAHD areas (Fig. 9), further heightening interest in these OTUs as contributing causes of early TAHD lesions that may exhibit some variation across TAHD’s range. One bacterial species included in these ASVs, *M. caseolyticus*, has been linked to dermatitis in donkeys (63) and dogs (64), and further investigation is needed to determine its role, if any, in early TAHD lesions.

*Fusobacterium* and several other opportunistic bacterial pathogens were associated with TAHD-positive lesions (Fig. 4 and 5; Fig. S3 and S4); however, the role of most of these genera in the development and progression of TAHD remains elusive. Similar to what has been reported in BDD (10, 23, 47), enrichment of *Fusobacterium* and numerous other anaerobes, including Clostridiales Family *XI*, Clostridiales Family *XIII*, *Actinomycetaceae* (including *T. pyogenes*), *Filifactor*, and *P. uenonis*, may imply a distinct shift in the bacterial community associated with TAHD. Because these organisms are typically members of the mammalian or soil microbiomes, they may also act as opportunistic pathogens. Detection and abundance of *Fusobacterium* were positively correlated with both *Treponema* and *Mycoplasma* (Fig. 6) and with numerous other TAHD-associated OTUs, suggesting that *Fusobacterium* may be a member of a core group of bacteria that are strongly associated with TAHD. Furthermore, detection of *Fusobacterium* was strongly associated with TAHD-positive lesions, best demonstrated by *Fusobacterium* having the highest odds ratio of all OTUs associated with TAHD-positive lesions (OR = 82, 95% CI = 14.5, 1,562.0; Fig. 4). *Fusobacterium*, *F. necrophorum*, and family *Fusobacteriaceae* have been associated with TAHD and a variety of foot diseases in livestock (4, 6, 10, 65, 66), suggesting that *Fusobacterium* may play a role in the development of pododermatitis.

Our findings highlight the importance of histologic evaluation for accurate TAHD diagnosis, as gross hoof deformities are not pathognomonic, and gross grade may not be the most representative of the microenvironment being examined during microbiome assessment. While TAHD was diagnosed with histopathology in the majority of hooves we classified as gross grades II–IV, some graded lesions were determined to be non-TAHD lesions (28%; Table S2), a heterogeneous group of histologically abnormal hooves that likely include variant normal hooves and hooves with inflammation due to other infectious or non-infectious processes. Moreover, 19% of grade I or grade II lesions were ultimately diagnosed on histologic examination as TAHD-negative tissues (Table S2). These results confirm that visual inspection alone does not accurately classify the TAHD status of some hooves.

We identified a few significant differences in the microbial communities associated with TAHD-positive lesions from endemic versus sporadic areas (Fig. 9; Fig. S7 and S8). Most of these differences were at the ASV level (Fig. 9) in OTUs that were not associated with TAHD-positive lesions and were not related to TAHD-associated families or genera identified by this study (Fig. 4 and 5; Fig. S3 and S4). These results suggest that regional differences in TAHD-associated pathogens may not be the driving force behind regional variation in TAHD’s prevalence and that environmental and host factors may play a more significant role in the observed differences. The few significant differences identified on comparison of the microbial communities in TAHD-positive lesions from endemic and sporadic areas may be due to limited sample sizes, regional variation in the cutaneous or soil microbiomes, or differences in lesion chronicity and severity. While two spirochete ASVs were identified as significantly associated with TAHD-positive lesions from endemic or sporadic areas, these differences were based on detection in a minority of TAHD-positive cases overall (unidentified *Spirochaetaceae* sp66603, 8/51; *T. phagedenis*, 19/51; Fig. 9), and further investigation of these ASVs in future studies is warranted to determine their significance.

Overall, our findings suggest that TAHD is a polymicrobial disease. OTUs within families *Spirochaetaceae* and *Mycoplasmataceae* were consistently associated with TAHD-positive and more severe histologic lesions, with strong positive correlations identified between the detection and abundance of genera *Treponema*, *Mycoplasma*, and unidentified *Spirochaetaceae*, consistent with a synergistic polybacterial infection. These OTUs should be targeted for the development of specific and sensitive diagnostic tests to supplement the current histologic diagnosis. Concurrent assessment of histologic lesion severity in combination with direct detection and localization of these putative pathogens in histologic sections with immunohistochemistry or *in situ* hybridization will be useful to confirm the microbial drivers of TAHD. While we identified significant bacterial enrichment of a variety of OTUs in TAHD-positive lesions, TAHD’s etiology and pathogenesis are likely due to a combination of microbial dysbiosis, immune modulation, environmental factors, and pathogen interactions. Understanding these dynamics requires more in-depth studies of bacterial-host interactions, microbial ecology, and prospective assessment of TAHD lesion progression.

## Data Availability

V3-V4 16S rRNA gene amplicon sequences are available at the National Center for Biotechnology Information, National Library of Medicine accession PRJNA1240626. Additional supplemental data available include abundance at various taxonomic levels with sample metadata and data from 16S V3-V4 rRNA gene amplicon sequencing read processing for 132 cases (Data set S1), the comparison of diversity indices (Data set S2), differential abundance analyses (Data set S3 to S5), and V3-V4 sequences and sequence identities for all Spirochaetaceae and Mycoplasma ASVs detected within samples (Data set S6). Data organization and statistical analyses were conducted using RStudio (v. 2024.09.0+375), R v. 4.4.1, and Microsoft Excel. OTU relative abundances were calculated at the family, genus, and ASV level following agglomeration with the "tax_glom" function in the phyloseq R package (67). Additional packages for data analysis and figure generation included tidyverse (68), RColorBrewer (69), and readxl (70).

## SUPPLEMENTAL MATERIAL

Data Set S1 aem.00888-25-s0001.xlsx 16S V3-V4 rRNA amplicon sequencing read processing data for 132 samples; metadata and abundance tables for 129 samples included in analyses of 16S rRNA gene amplicon sequencing data at the family, genus, and ASV level.

Data Set S2 aem.00888-25-s0002.xlsx Summary of the results of comparisons of Shannon diversity indices and Bray-Curtis dissimilarity.

Data Set S3 aem.00888-25-s0003.xlsx Summary of the results of the differential abundance analyses by TAHD diagnosis at the family, genus, and ASV levels.

Data Set S4 aem.00888-25-s0004.xlsx Summary of the results of the differential abundance analyses by histologic lesion category at the family, genus, and ASV levels.

Data Set S5 aem.00888-25-s0005.xlsx Summary of the results of the differential abundance analyses for TAHD-positive lesions by TAHD prevalence at the family, genus, and ASV levels.

Data Set S6 aem.00888-25-s0006.xlsx V3-V4 16S rRNA amplicon sequences and sequence identities for all Spirochaetaceae and Mycoplasmataceae ASVs detected within samples.

Supplemental figures and tables aem.00888-25-s0007.pdf Figures S1 to S8; Tables S1 and S2.

ASM does not own the copyrights to Supplemental Material that may be linked to, or accessed through, an article. The authors have granted ASM a non-exclusive, world-wide license to publish the Supplemental Material files. Please contact the corresponding author directly for reuse.
