# Supplementary material for: Bacterial community analysis of treponeme-associated hoof disease in free-ranging elk (Cervus canadensis): evidence for a polybacterial etiology with geographic consistency
Source: Appl Environ Microbiol. 2025 Oct 3;91(11):e00888-25. doi: 10.1128/aem.00888-25 (PMC12628766; doi:10.1128/aem.00888-25)
Supplement: Supplemental figures and tables — Figures S1 to S8; Tables S1 and S2. [file aem.00888-25-s0007.pdf]

SUPPLEMENTAL FIGURES

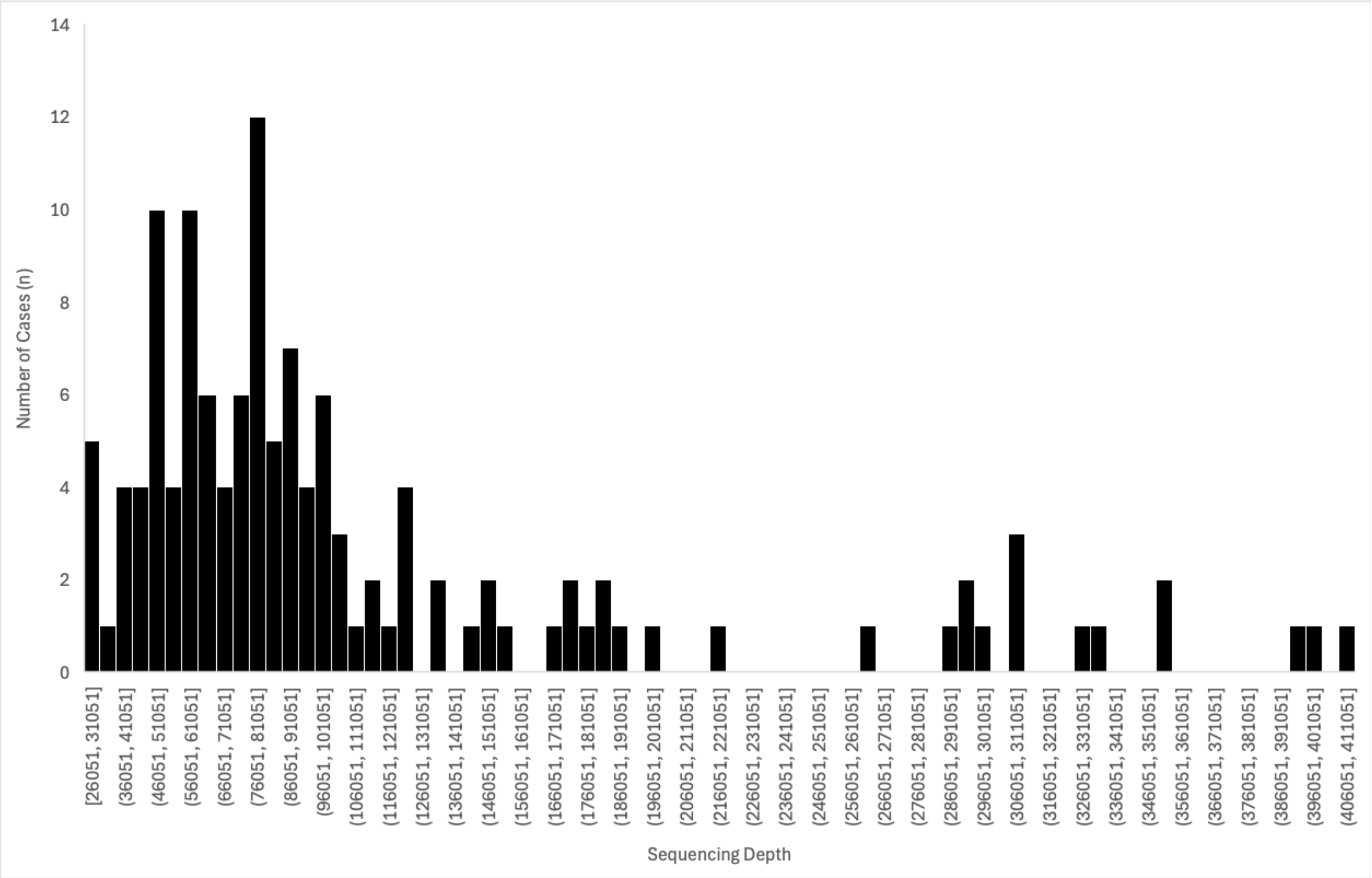

***Figure S1*: Sequencing depth for 16S V3-V4 rRNA amplicon sequencing of extracted genomic DNA from all elk interdigital skin samples.** Histogram of sequencing depth following post-sequencing processing and removal of non-bacterial reads in class Chloroplast and family *Mitochondria* for 129 cases included in analyses of 16S rRNA gene amplicon sequencing data.

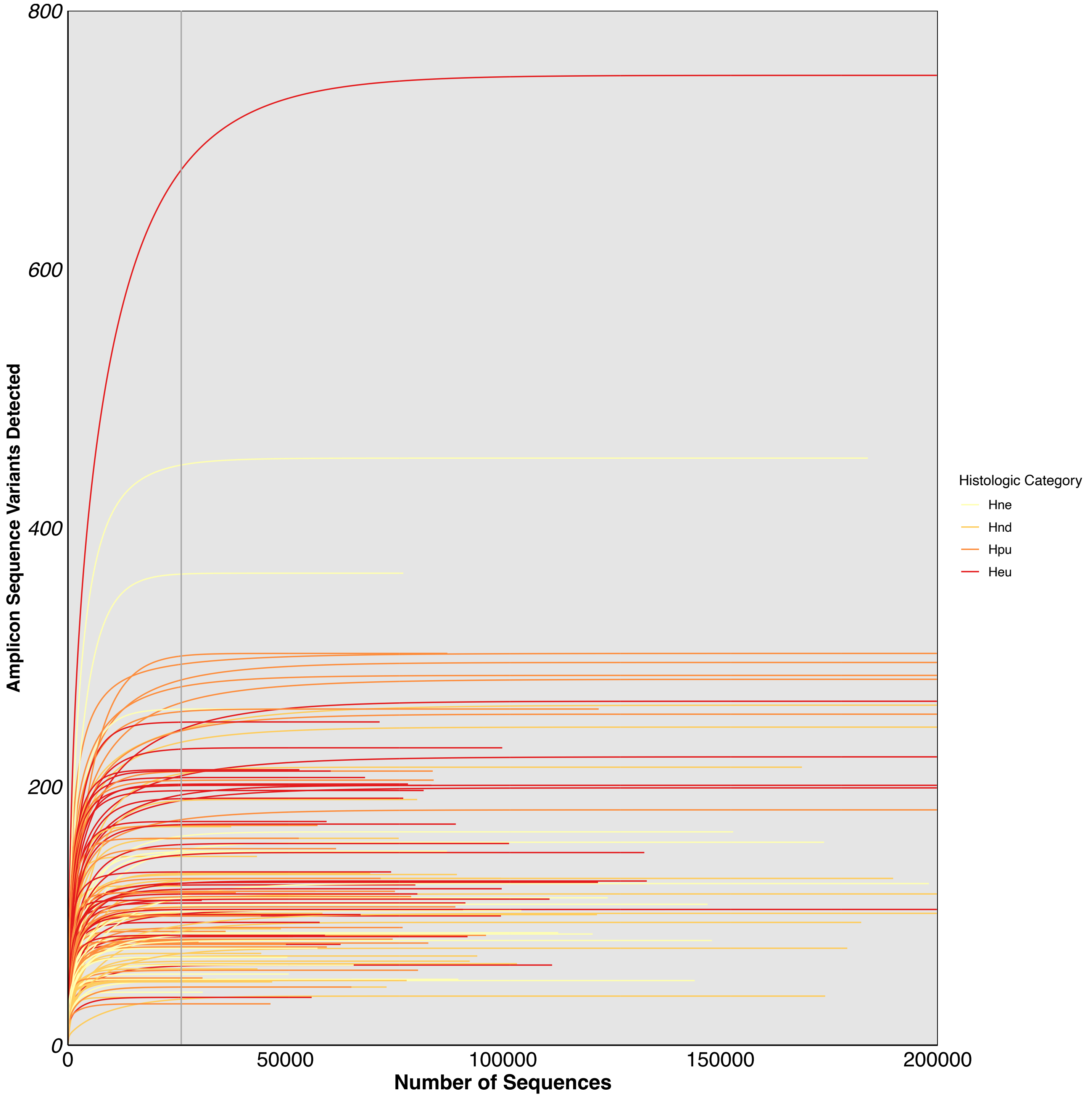

***Figure S2*: Rarefaction curves for 16S V3-V4 rRNA amplicon sequencing of extracted genomic DNA from all elk interdigital skin samples**. Rarefaction curves generated with a step of 200 (*rarecurve*, *vegan*) following post-sequencing processing, removal of all sequences in class Chloroplast and family *Mitochondria*, and agglomeration to the amplicon sequence variant level for 129 cases included in analyses of 16S amplicon sequencing data.

Associated Families	Percent of Samples with OTU Detected (Detection Frequency)		Log-fold Change in Abundance	Proportion Test	Odds of TAHD-Positive vs. TAHD-Negative Diagnosis	
	**TAHD-Positive** n=51	**TAHD-Negative** n=21	**TAHD-Positive vs. TAHD-Negative**	**Adjusted P-Value**	**Odds Ratio**	(95% Confidence Interval)
*Mycoplasmataceae**	78%	0%		< 0.001		
*Spirochaetaceae**	80%	29%	3.70	0.004	**10.3**	(3.3, 35.5)
Clostridiales Family *XIII**	84%	24%	3.30	< 0.001	**17.2**	(5.2, 66.5)
*Fusobacteriaceae**	80%	5%	2.73	< 0.001	**82.0**	(14.5, 1562.0)
*Enterobacteriaceae**	59%	10%	2.57	0.012	**13.6**	(3.4, 91.1)
*Peptococcaceae**	78%	19%	2.49	< 0.001	**15.5**	(4.7, 62.9)
Moraxellaceae	90%	86%	2.38	1.000	**1.5**	(0.3, 6.9)
*Actinomycetaceae**	92%	29%	2.13	< 0.001	**29.4**	(8.0, 134.6)
Peptostreptococcaceae	100%	62%	1.88	0.001		
Lachnospiraceae	75%	38%	1.79	0.223	**4.8**	(1.6, 14.6)
Xanthomonadaceae	69%	33%	1.50	0.373	**4.4**	(1.5, 13.6)
Bacteroidaceae	61%	10%	1.45	0.009	**14.7**	(3.7, 98.9)
Campylobacteraceae	69%	38%	1.23	0.718	**3.6**	(1.3, 10.7)
Coriobacteriaceae	78%	29%	1.10	0.008	**9.1**	(3.0, 31.0)
Corynebacteriaceae	100%	100%	-1.25			
Aerococcaceae	94%	100%	-2.11	1.000		

(A)

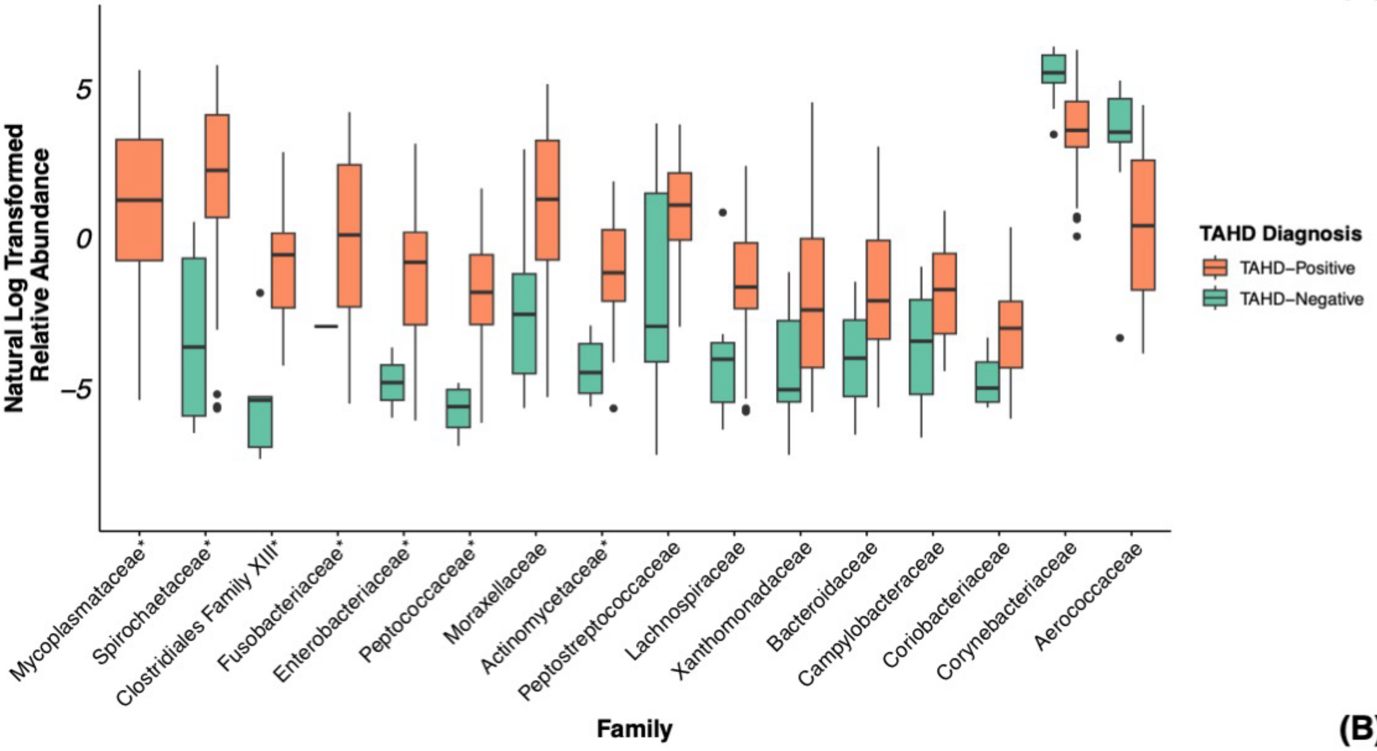

(B)

***Figure S3*: Families associated with treponeme-associated hoof disease (TAHD)-positive lesions or TAHD-negative tissues.** (A) Heatmap of detection frequencies for families associated with TAHD-positive lesions or TAHD-negative tissues with additional significance criteria. (B) Boxplot summarizing natural log transformed relative abundance for associated families by TAHD diagnosis with boxplots (median, 25th quartile, and 75th quartile), whiskers (1.5x IQR), and outliers (black dots, > 1.5x IQR). *Family met all established significance criteria.

Associated Amplicon Sequence Variants (ASVs) in Clostridiales Family *XI* or *XIII*	Percent of Samples with OTU Detected (Detection Frequency) TAHD-Positive n=51	TAHD-Negative n=21	Proportion Test Adjusted P-Value
*Peptoniphilus indolicus**	82%	0%	< 0.001
Unidentified *Clostridiales* Family XIII sp31685*	80%	0%	< 0.001
Unidentified *Anaerococcus**	71%	0%	< 0.001
Ezakiella sp31234*	71%	0%	< 0.001
Gallicola sp31244*	65%	0%	< 0.001
Tissierella sp31385*	65%	0%	< 0.001
Unidentified *Clostridiales* Family XIII sp31686*	65%	0%	< 0.001
Unidentified *Clostridiales* Family XI sp31397*	61%	0%	< 0.001
Parvimonas sp31278*	61%	0%	< 0.001
Gallicola sp31243*	53%	0%	0.004
*Peptoniphilus coxii**	51%	0%	0.006

(A)

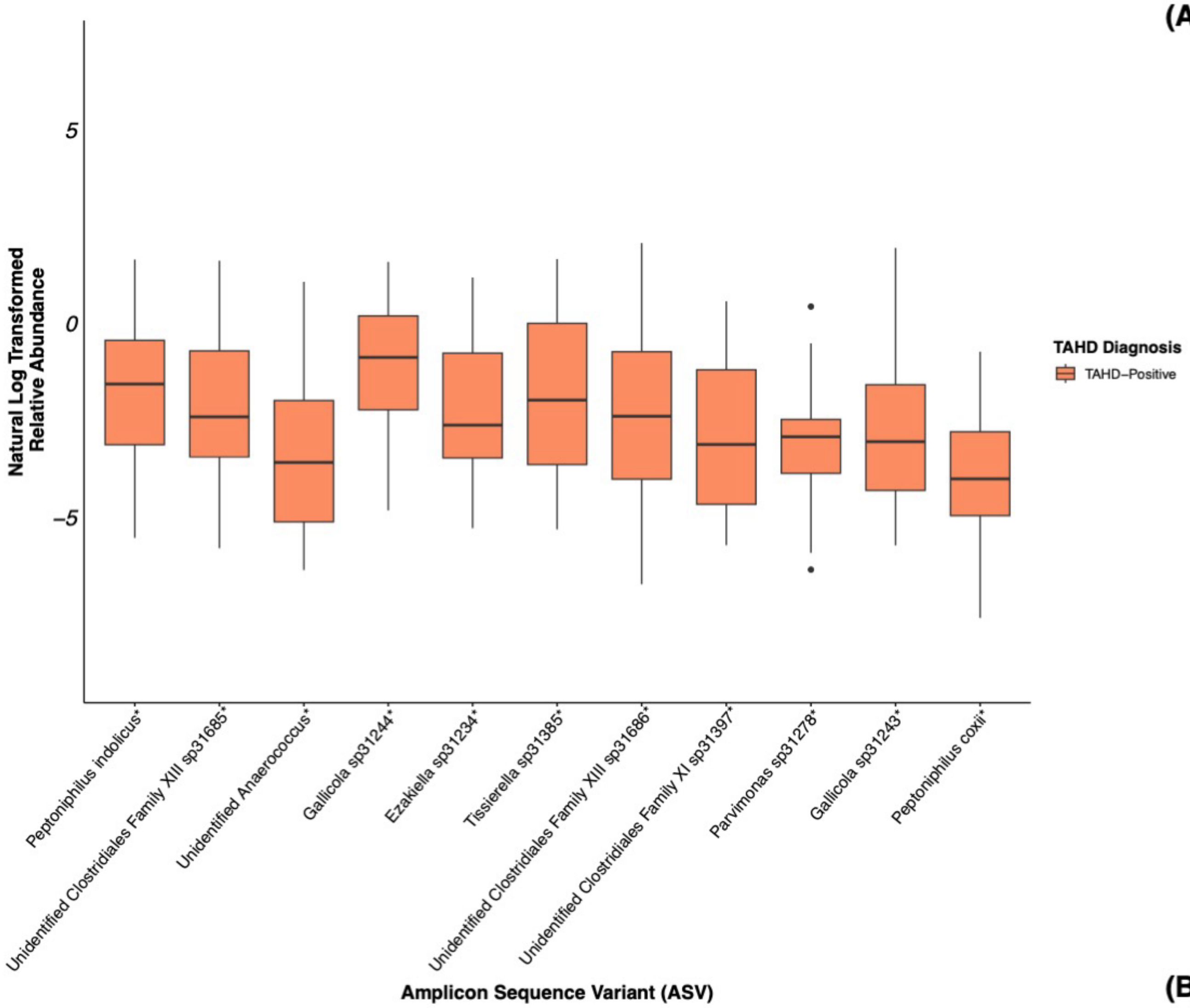

(B)

***Figure S4*: Amplicon sequence variants (ASVs) in Clostridiales Family *XI* or *XIII* associated with treponeme-associated hoof disease (TAHD)-positive lesions and TAHD-negative tissues.** (A) Heatmap of detection frequencies for ASVs associated with TAHD-positive lesions or TAHD-negative tissues with additional significance criteria. (B) Boxplot summarizing natural log transformed relative abundance for associated ASVs by TAHD diagnosis with boxplots (median, 25th quartile, and 75th quartile), whiskers (1.5x IQR), and outliers (black dots, > 1.5x IQR). *ASV met all established significance criteria.

Associated Families	Percent of Samples with OTU Detected (Detection Frequency): Normal Epidermis (H_{ne}) n=21	Intracorneal Necrotic Cellular Debris (H_{nd}) n=34	Pustular Dermatitis (H_{pu}) n=37	Erosive to Ulcerative Dermatitis (H_{eu}) n=37	Log-fold Change in Abundance: H_{pu} vs. H_{ne}	H_{eu} vs. H_{ne}	Odds of More Severe vs. Less Severe Disease: **Odds Ratio** (95% Confidence Interval)	
*Mycoplasmataceae**	0%	15%	46%	76%			**12.6**	(5.8, 27.6)
*Spirochaetaceae**	29%	35%	54%	76%	2.81	3.95	**3.9**	(2.0, 7.5)
Clostridiales Family XIII*	24%	26%	59%	92%	2.33	3.29	**9.6**	(4.6, 20.1)
*Fusobacteriaceae**	5%	15%	43%	84%	1.98	2.96	**15.5**	(6.9, 34.7)
*Moraxellaceae**	86%	53%	84%	89%		2.79	**2.1**	(> 1.0**, 4.2)
*Actinomycetaceae**	29%	38%	70%	97%		2.62	**10.0**	(4.7, 21.1
*Peptococcaceae**	19%	26%	43%	84%	2.35	2.58	**7.1**	(3.5, 14.4)
Aerococcaceae	100%	88%	100%	92%		-2.16	**0.8**	(0.2, 3.2)

(A)

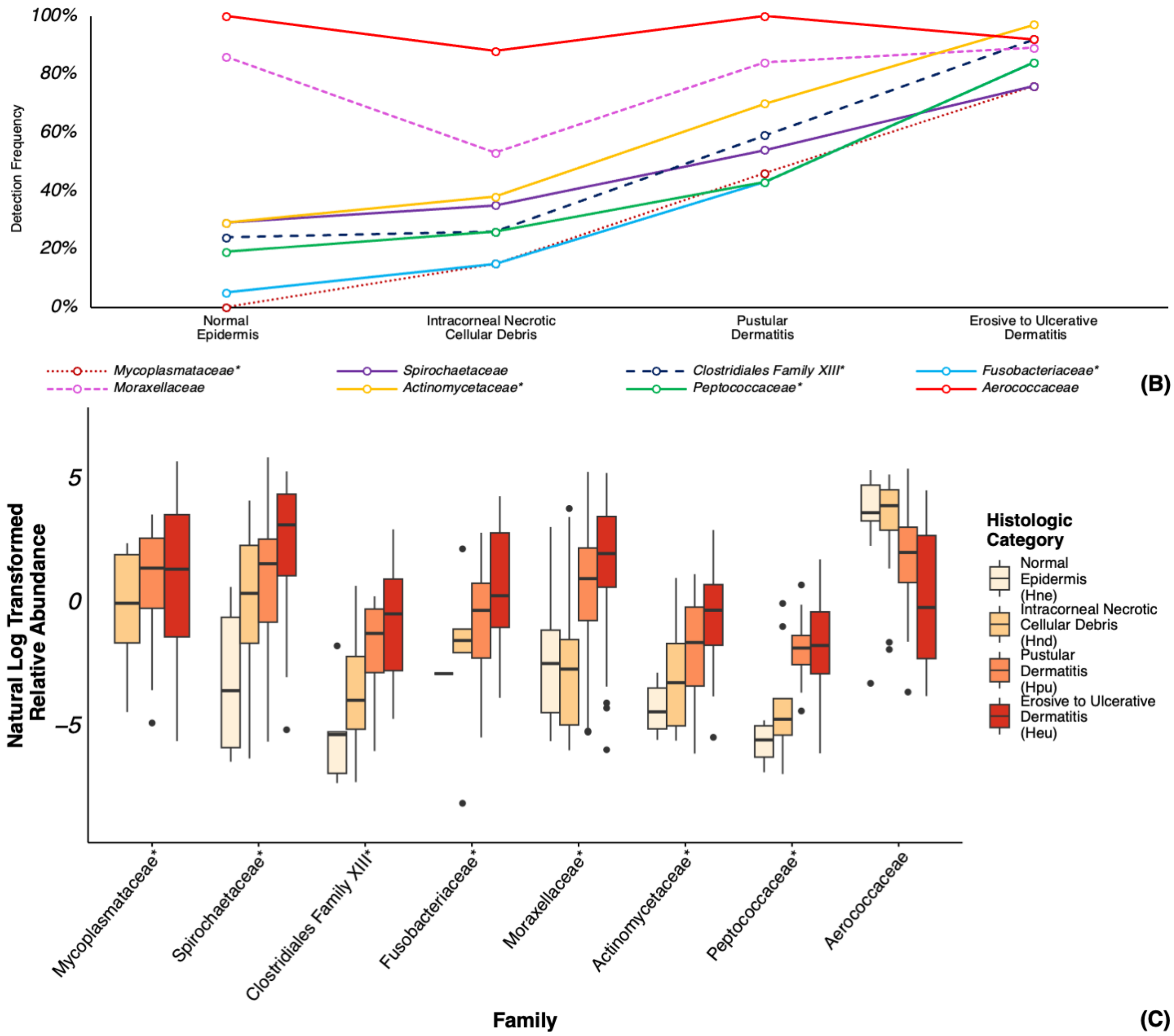

Figure S5: **Families associated with histologic lesion categories.** (A) Heatmap of detection frequencies of all samples (TAHD-positive, TAHD-negative, and non-TAHD lesions) for families associated with both histologic lesion categories and TAHD diagnoses (TAHD-positive lesions or TAHD-negative tissues) with additional significance criteria. (B) Boxplot summarizing natural log transformed relative abundance for associated families by histologic category with boxplots (median, 25th quartile, and 75th quartile), whiskers (1.5x IQR), and outliers (black dots, > 1.5x IQR). *Family met all established significance criteria. **Lower limit of 95% confidence interval for *Moraxellaceae* is 1.004.

Associated Amplicon Sequence Variants (ASVs) in Clostridiales Family *XI* or *XIII*	Percent of Samples with OTU Detected (Detection Frequency)				Odds of More Severe vs. Less Severe Disease	
	Normal Epidermis (H_{ne}) n=21	Intracorneal Necrotic Cellular Debris (H_{nd}) n=34	Pustular Dermatitis (H_{pu}) n=37	Erosive to Ulcerative Dermatitis (H_{eu}) n=37	Odds Ratio (95% Confidence Interval)	
*Peptoniphilus indolicus**	0%	6%	46%	86%	**33.2**	(12.8, 86.2)
Unidentified Clostridiales Family XIII sp31685*	0%	9%	51%	81%	**20.5**	(8.8, 48.1)
Gallicola sp31244*	0%	6%	30%	76%	**21.5**	(8.8, 52.4)
Ezakiella sp31234*	0%	9%	38%	70%	**13.3**	(5.9, 29.8)
Tissierella sp31385*	0%	3%	35%	68%	**16.1**	(6.9, 37.6)
Unidentified *Clostridiales* Family *XIII* sp31686*	0%	6%	30%	68%	**15.1**	(6.4, 35.5)
Unidentified *Anaerococcus**	0%	12%	43%	65%	**9.3**	(4.3, 19.9)
Unidentified *Clostridiales* Family *XI* sp31397*	0%	6%	32%	65%	**13.0**	(5.7, 29.9)
Parvimonas sp31278*	0%	12%	30%	65%	**10.4**	(4.7, 23.2)
Gallicola sp31243*	0%	9%	35%	57%	**8.3**	(3.8, 18.0)
*Peptoniphilus coxii**	0%	0%	22%	57%	**17.2**	(6.7, 44.5)

(A)

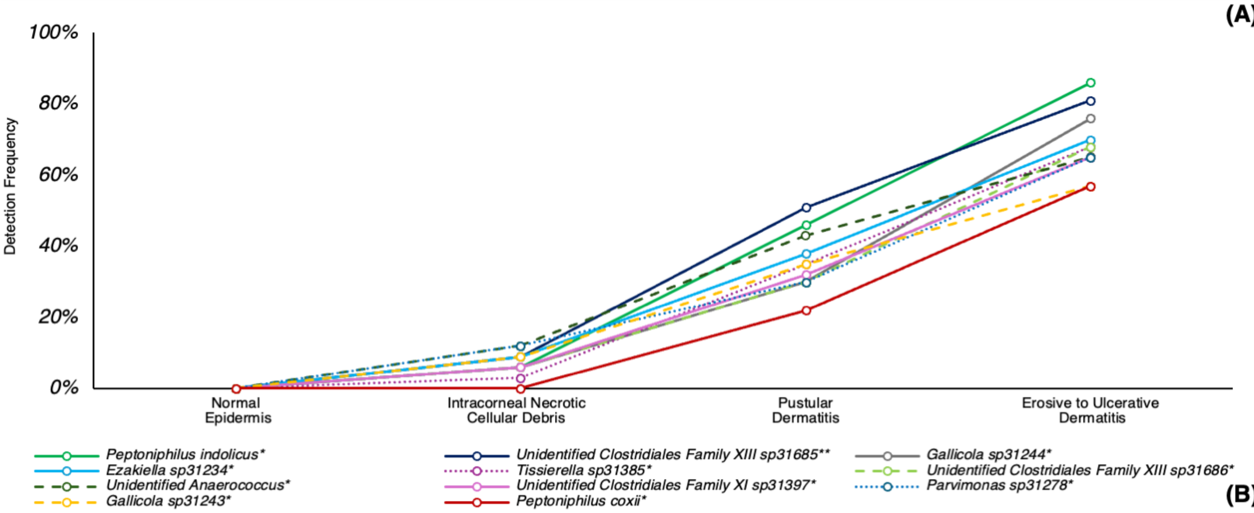

(B)

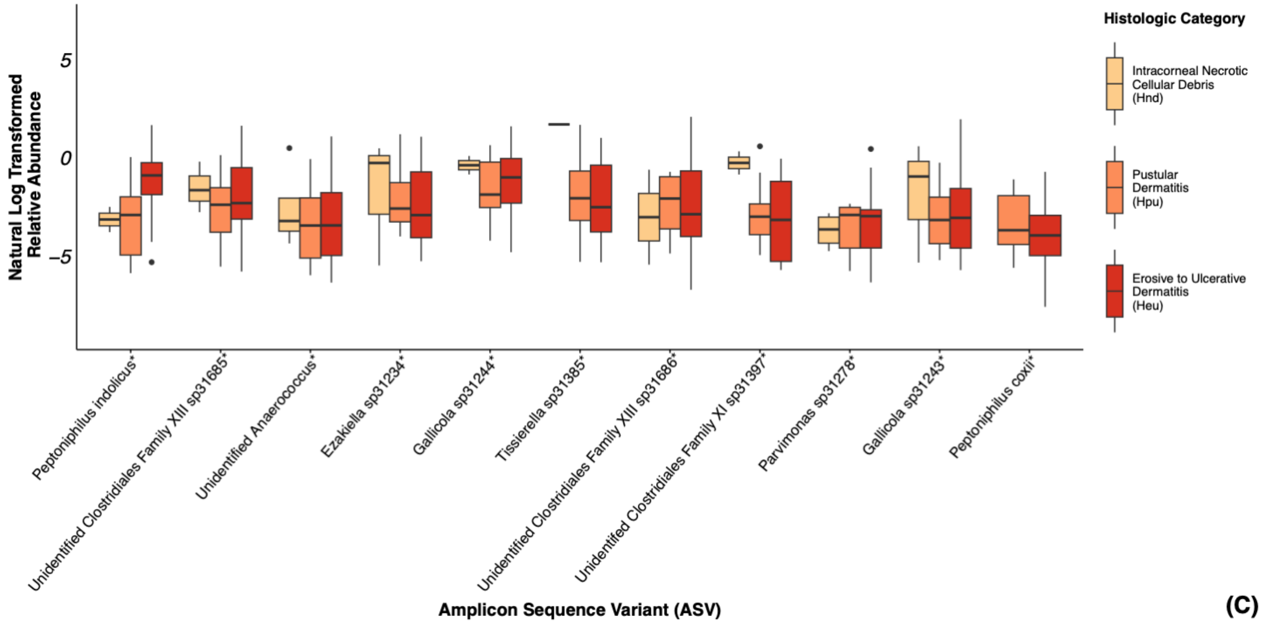

(C)

***Figure S6*: Amplicon sequence variants (ASVs) in Clostridiales Family *XI* or *XIII* associated with histologic lesion categories.** (A) Heatmap of detection frequencies of all samples (TAHD-positive, TAHD-negative, and non-TAHD lesions) for ASVs associated with both histologic lesion categories and TAHD diagnoses (TAHD-positive lesions or TAHD-negative tissues) with additional significance criteria. (B) Boxplot summarizing natural log transformed relative abundance for associated ASVs by histologic lesion category with boxplots (median, 25th quartile, and 75th quartile), whiskers (1.5x IQR), and outliers (black dots, > 1.5x IQR). *ASV met all established significance criteria.

Associated Families	*Percent of Samples with OTU Detected (Detection Frequency)* Endemic Areas n=39	Sporadic Areas n=12	*Log-fold Change in Abundance (LFC)* TAHD-Positive Lesions from Endemic vs. Sporadic Areas	*Proportion Test* ***Adjusted P-Value***
Deinococcaceae	36%	75%	-1.83	0.850
*Dermabacteraceae**	13%	75%	-2.03	0.006
Dermatophilaceae	41%	67%	-2.05	1.000
Propionibacteriaceae	74%	100%	-2.16	1.000
Staphylococcaceae	92%	100%	-2.83	1.000
Aerococcaceae	92%	100%	-2.84	1.000

(A)

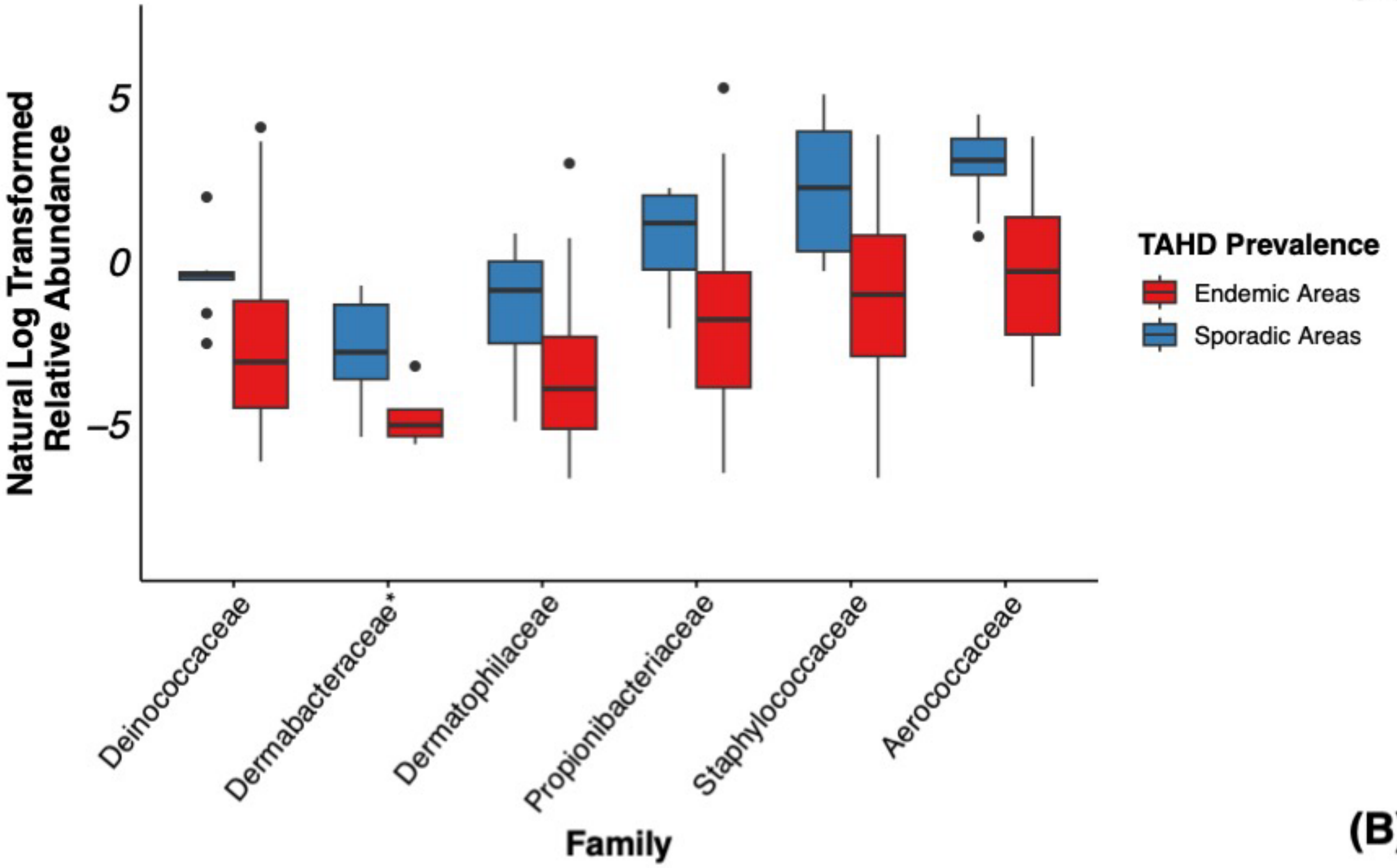

(B)

***Figure S7*: Families associated with treponeme-associated hoof disease (TAHD)-positive lesions from endemic versus sporadic areas.** (A) Heatmap of detection frequencies for families associated with TAHD-positive lesions from endemic or sporadic areas with additional significance criteria. (B) Boxplot summarizing natural log transformed relative abundance for families associated with TAHD-positive lesions from endemic or sporadic areas with boxplots (median, 25th quartile, and 75th quartile), whiskers (1.5x IQR), and outliers (black dots, > 1.5x IQR). *Family met all established significance criteria.

Associated Genera	Percent of Samples with OTU Detected (Detection Frequency) Endemic Areas n=39	Sporadic Areas n=12	Log-fold Change in Abundance (LFC) TAHD-Positive Lesions from Endemic vs. Sporadic Areas	Proportion Test Adjusted P-Value
Arcanobacterium	23%	92%	-1.73	0.004
Deinococcus	36%	75%	-1.83	0.850
Brachybacterium	10%	75%	-1.93	0.002
Facklamia	67%	100%	-2.31	0.999
Ignavigranum	82%	100%	-2.76	1.000
Macrococcus	90%	100%	-2.84	1.000

(A)

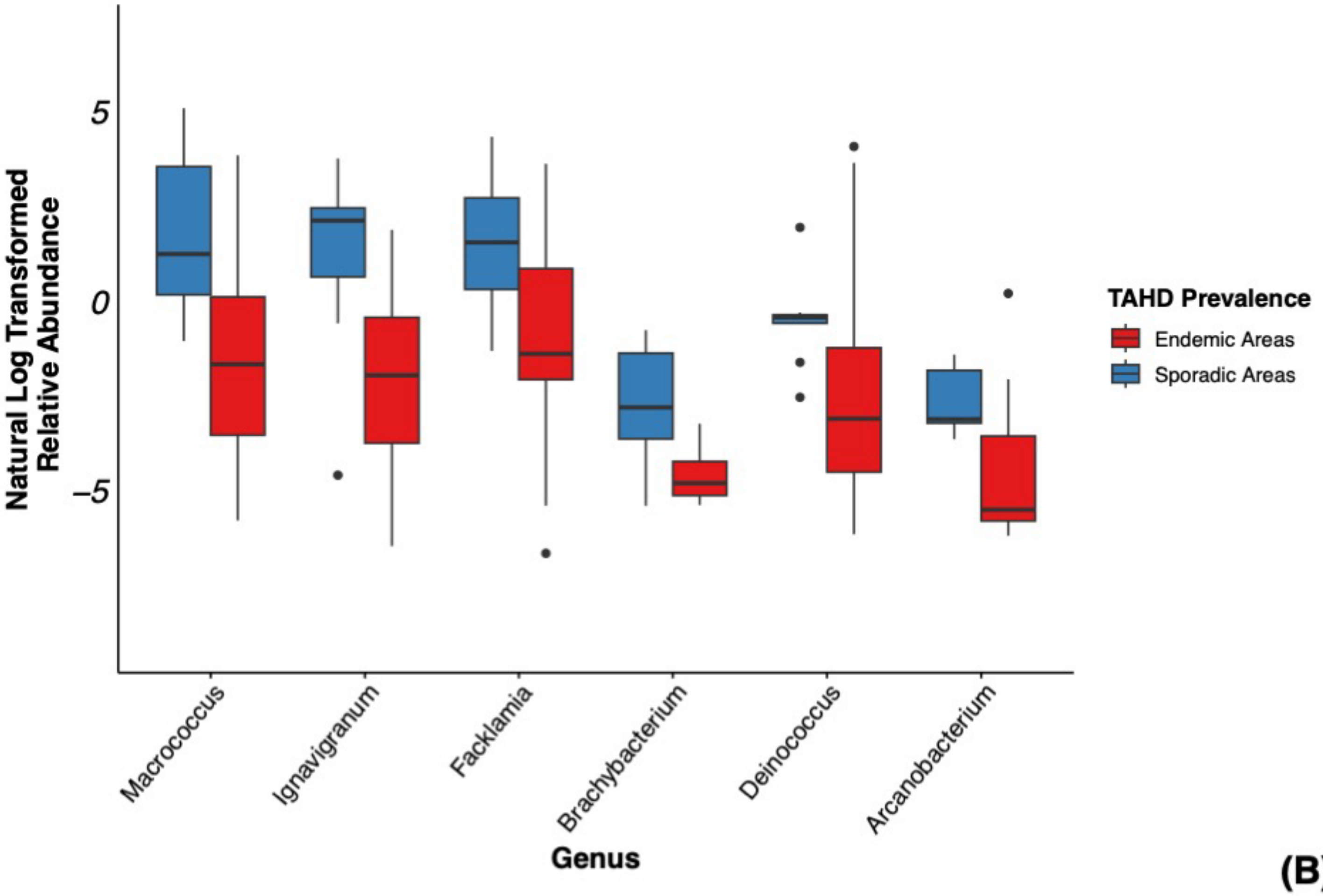

(B)

***Figure S8*: Genera associated with treponeme-associated hoof disease (TAHD)-positive lesions from endemic versus sporadic areas.** (A) Heatmap of detection frequencies for genera associated with TAHD-positive lesions from endemic or sporadic areas with additional significance criteria. (B) Boxplot summarizing natural log transformed relative abundance for genera associated with TAHD-positive lesions from endemic or sporadic areas with boxplots (median, 25th quartile, and 75th quartile), whiskers (1.5x IQR), and outliers (black dots, > 1.5x IQR). *Genus met all established significance criteria.

SUPPLEMENTAL TABLES

Most Severe Gross Grade (adapted from Han *et al.*, 2019)	Histologic Category: Normal Epidermis (H_{ne}) n=21	Intracorneal Necrotic Cellular Debris (H_{nd}) n=34	Pustular Dermatitis (H_{pu}) n=37	Erosive to Ulcerative Dermatitis (H_{eu}) n=37	*Lesions with Neutrophilic Inflammation ($H_{pu} + H_{eu}$) n=74*	Total n=129
**Grade 0**	17	20	10	2	*12*	**49**
**Grade I**	3*	6	4	3	*7*	**16**
**Grade II**	1*	1	4	1	*5*	**7**
**Grade III**	0*	1	3	7	*10*	**11**
**Grade IV**	0*	0	9	24	*33*	**33**
Not Graded/ Incomplete Grading	*0**	*6*	*7*	*0*	*7*	***13***

***Table S1*: Treponeme-associated hoof disease (TAHD) gross lesion grades for elk cases with normal and abnormal hooves by histologic lesion category.** Gross lesion grades for TAHD from 129 elk sampled for 16S rRNA gene amplicon sequencing analyses by histologic lesion category. Gross grades were defined as follows (adapted from Han *et al*., 2019): Grade 0: Hooves free of significant gross abnormalities; Grade I: Hoof with presumptive cutaneous erosion or ulceration of the coronary band, interdigital skin, and/or heel bulb without involvement of the sole or deformity of the hoof capsule; Grade II: Grade I hoof lesion with undermining of the heel bulb and/or hoof capsule; Grade III: Grade I hoof lesion with ulceration of the sole of the hoof; Grade IV: Grade II or III hoof lesions with broken or sloughed hoof capsules. *Cases excluded if histologic lesions deemed non-representative of significant hoof abnormalities.

Most Severe Gross Grade (adapted from Han *et al.*, 2019)	TAHD-Positive All n=51	*Endemic n=39*	*Sporadic n=12*	TAHD-Negative n=21	Non-TAHD Lesions n=57	Total **n=129**
**Grade 0**	4	*1*	*3*	17	28	**49**
**Grade I**	4	*4*	*0*	3*	9	**16**
**Grade II**	4	*1*	*3*	1*	2	**7**
**Grade III**	8	*3*	*5*	0*	3	**11**
**Grade IV**	28	*27*	*1*	0*	5	**33**
Not Graded/ Incomplete Grading	3	*3*	*0*	*0**	*10*	***13***

***Table S2*: Treponeme-associated hoof disease (TAHD) gross lesion grades for elk cases with normal and abnormal hooves by TAHD diagnosis**. Gross lesion grades (adapted from Han *et al.*, 2019; see *Table S1* legend for grade definitions) for TAHD from 129 elk sampled for 16S rRNA gene amplicon sequencing analyses by TAHD diagnosis. Gross grades were defined as follows (adapted from Han *et al.*, 2019): Grade 0: Hooves free of significant gross abnormalities; Grade I: Hoof with presumptive cutaneous erosion or ulceration of the coronary band, interdigital skin, and/or heel bulb without involvement of the sole or deformity of the hoof capsule; Grade II: Grade I hoof lesion with undermining of the heel bulb and/or hoof capsule; Grade III: Grade I hoof lesion with ulceration of the sole of the hoof; Grade IV: Grade II or III hoof lesions with broken or sloughed hoof capsules. *Cases excluded if histologic lesions deemed non-representative of significant hoof abnormalities.
